# An evaluation of the efficacy of single-echo and multi-echo fMRI denoising strategies

**DOI:** 10.1162/NETN.a.547

**Published:** 2026-04-22

**Authors:** Toby Constable, Jeggan Tiego, Kane Pavlovich, Arshiya Sangchooli, Priscilla Thalenberg Levi, Bree Hartshorn, Jessica Kwee, Kate Fortune, Kate Thompson, Sam Brown, James McLauchlan, Nancy Ong Tran, Rebecca O’Neill, Mark A. Bellgrove, Alex Fornito

**Affiliations:** School of Psychological Sciences, Turner Institute for Brain and Mental Health, and Monash Biomedical Imaging, Monash University, Melbourne, Australia

**Keywords:** fMRI, Preprocessing, Multi-echo, Kernel ridge regression, Methods, Motion

## Abstract

Resting-state functional magnetic resonance imaging (rsfMRI) is widely used to study brain-wide functional connectivity (FC). However, the resulting signals are highly noise sensitive, and the best strategies for mitigating this noise remains unclear. In 358 healthy individuals, we compared 60 multi-echo (ME) and 30 single-echo (SE) rsfMRI preprocessing pipelines across six measures of data quality and associated effect sizes in FC-based prediction models of personality and cognition (cross-validated kernel ridge regression). ME pipelines generally outperformed SE pipelines, but no single pipeline excelled at both denoising and behavioral prediction. Using a heuristic scheme to rank pipelines across benchmarks, ME optimum combination (OC) with ME independent component analysis (ICA), ICA-FMRIB’s ICA-based Xnoiseifier (FIX), and with head motion, cerebrospinal fluid, and white matter and gray matter signal regression, performed best when only considering denoising efficacy metrics. ME OC with ICA-FIX and head motion parameter regression performed best when only considering behavioral prediction results. ME OC with Automatic Removal of Motion Artifacts (AROMA) ICA, head motion parameter regression and Regressor Interpolation at Progressive Time Delays (RIPTiDe) performed best when aggregating across all evaluation metrics. These results favor ME acquisitions but show that no single denoising pipeline should be considered optimal for all purposes.

## Author Summary

Resting-state fMRI is widely used to study brain connectivity but is plagued by diverse sources of noise. We evaluated novel and established fMRI denoising techniques across 90 preprocessing pipelines, including multi-echo and single-echo approaches, in 358 healthy adults. Pipelines were evaluated using established noise-detection heuristics and their ability to predict individual differences in cognition and personality using cross-validated kernel-ridge regression. Multi-echo pipelines generally outperformed single-echo approaches, but no single pipeline performed best across all benchmarks. Pipelines that maximized noise reduction tended to perform worse in behavioral prediction. Overall, our findings support the use of multi-echo acquisitions while demonstrating that no single denoising pipeline is optimal for all research purposes.

## AN EVALUATION OF THE EFFICACY OF SINGLE-ECHO AND MULTI-ECHO (ME) fMRI DENOISING STRATEGIES

Functional magnetic resonance imaging (fMRI) detects changes in blood (de)oxygenation to infer neuronal activity (Hillman, 2014; Ogawa et al., 1990). The resulting blood oxygenation level-dependent (BOLD) signal is heavily influenced by nonneuronal factors such as head motion and physiological noise, which can compromise measurement fidelity (Hillman, 2014; Liu, 2016; Ogawa et al., 1990) and thus complicate attempts to obtain reliable effect sizes for brain–behavior associations (Marek et al., 2022). This concern is particularly acute in the analysis of resting-state interregional functional connectivity (FC) estimates, which are notoriously susceptible to various sources of noise (Birn et al., 2014). Developing preprocessing strategies for more effective separation of signal from noise is essential for generating valid and replicable insights from these analyses.

Head motion constitutes one of the most influential sources of noise in BOLD data (Aquino et al., 2020; Ciric et al., 2017; Parkes et al., 2018; Power et al., 2012; Satterthwaite et al., 2013; Van Dijk et al., 2012). A simple strategy for addressing motion-related confounds in fMRI analyses involves regressing the six standard head motion parameters describing rotations and translations measured during image realignment, their quadratic expansion, first-order derivatives, and the quadratics of the derivatives from the BOLD signal using a procedure dubbed 24P regression (Satterthwaite et al., 2013). To further account for signal fluctuations of a nonneuronal origin, it is also common to remove signals averaged from the cerebrospinal fluid (CSF) and white matter (WM) tissue compartments (which we term 2P regression), optionally also in combination with their expansion and derivative terms (which we collectively term 8P regression; Aquino et al., 2020; Parkes et al., 2018). A common additional step involves performing global signal regression (GSR) or gray matter signal regression (GMSR), which involves removing the average signal taken from the entire brain or gray matter tissue compartment and, optionally, the corresponding expansion and derivative terms. Finally, some analyses involve censoring high-motion frames, as estimated using a measure of framewise displacement (FD; Ciric et al., 2017; Parkes et al., 2018; Power et al., 2012), either by removing those frames or using delta function regressors (Satterthwaite et al., 2013). In some cases, an interpolation is applied to replace the missing data (Power et al., 2013; Wilke & Baldeweg, 2019).

The GSR and GMSR procedures have attracted controversy. They both center the distribution of FC estimates on zero (Fox et al., 2009), giving rise to spurious anticorrelations (Murphy et al., 2009) and distorting group differences in FC estimates (Saad et al., 2012). The technique may also be overly aggressive, potentially removing signals of interest from the data (Chang & Glover, 2009). However, global signal fluctuations are highly correlated with nonneuronal physiological fluctuations (Power et al., 2017; Xifra-Porxas et al., 2024), and their removal improves the anatomical specificity of interregional FC patterns (Fox et al., 2009), strongly mitigates motion-related signal contamination (Ciric et al., 2017; Parkes et al., 2018), improves correlations between FC and behavioral measures (J. Li et al., 2019), and yields BOLD signals that correlate more strongly with underlying neuronal calcium dynamics (Matsui et al., 2016).

Alternative methods for addressing global signal artifacts (also called widespread signal deflections, or WSDs) that may address the documented limitations of GSR have been proposed. One approach, termed Diffuse Cluster Estimation and Removal (DiCER), uses an iterative clustering procedure to remove WSDs, demonstrating variable performance relative to GSR depending on the characteristics of the data and the specific parameters used in its implementation (Aquino et al., 2020; Pavlovich et al., 2024). Another approach uses temporal independent component analysis (ICA) to separate components that represent distinct WSDs (Glasser et al., 2019). Although this approach offers an elegant solution, it requires large amounts of data and is difficult to apply to many extant fMRI datasets. One method gaining popularity involves estimating spatially and temporally heterogeneous low-frequency oscillations (LFOs) that are unlikely to have a neural origin directly from BOLD time-series data using spatially dependent time-lagged regression models, as implemented in the Regressor Interpolation at Progressive Time Delays (RIPTiDE) algorithm (deB Frederick et al., 2012). LFOs predominantly occur on the hemodynamic rather than neural time scale and propagate through the brain’s vasculature, suggesting that they may originate from changes in arterial pressure (Tong et al., 2019). Recent work has found that RIPTiDE can mitigate spurious inflations of FC that occur as a function of time spent in the scanner (functional connectivity inflation [FCI]; Korponay et al., 2024). GSR/GMSR can also reduce FCI, but an advantage of the RIPTiDe algorithm is that it does not yield spurious anticorrelations by construction (Erdoğan et al., 2016).

Another popular class of denoising strategies relies on spatial independent component analysis (s-ICA), which is a dimensionality reduction technique that decomposes spatiotemporal fMRI signals into multiple sources (independent components [ICs]) with maximal spatial independence (McKeown et al., 2003). Each IC may represent noise, neuronal signal, or a mixture of both. These components can be classified and used to selectively remove contributions from spatially structured noise signals. For instance, ICA-based Automatic Removal of Motion Artifacts (ICA-AROMA) is a widely used automatic denoising method that classifies components as either signal or noise based on several data-driven heuristics, under the assumption that true signal components typically exhibit low-frequency power, regular oscillations, and predominantly localize to gray matter (Griffanti et al., 2017; Pruim et al., 2015). ICA-AROMA thus identifies noise components based on high-frequency content, correlation with head motion realignment parameters, and activity near the edges of the brain or within the CSF (Pruim et al., 2015).

A more elaborate ICA-based denoising technique is Functional Magnetic Resonance Imaging of the Brain’s ICA-based X-noiseifier (ICA-FIX; Salimi-Khorshidi et al., 2014). This method first involves hand-classifying ICs as either “signal” or “noise” in a training set derived from a larger data pool. These labels then serve as a “ground truth” to train a classifier for automatically rejecting noise components for the rest of the dataset. This approach allows for more tailored classifications given the particulars of the data by leveraging human expertise to improve the denoising process (Griffanti et al., 2017). Signal components can be manually classified according to similar heuristics used by ICA-AROMA (e.g., high-frequency power, irregular oscillatory patterns, poor localization to gray matter), but can also include more complex spatial and/or temporal properties that ICA-AROMA may miss (Griffanti et al., 2017).

A third ICA-based approach requires a multi-echo (ME) acquisition acquisition. Unlike single-echo (SE) acquisitions, ME fMRI captures multiple images per radiofrequency (RF) pulse, each with varying contrast levels (Kundu et al., 2012, 2017; Lynch et al., 2021; Posse et al., 1999). Early echoes exhibit higher signal intensity and lower dropout due to their temporal proximity to the RF pulse, while later echoes provide greater tissue contrast but lower signal intensity, except for tissues with slower decay rates (Kundu et al., 2017). Applying a weighted average across each echo yields an optimally combined time series in which signal-quality and tissue-contrast ratios are maximized and signal dropout is minimized. BOLD and non-BOLD signals decay in intensity after each RF pulse at a known T_2_* rate, which can be compared with empirical T_2_* measures. When combined with s-ICA, ME acquisition can aid in distinguishing signal from noise based on the T_2_* of each IC. Comparing the relative fits of known BOLD and non-BOLD T_2_* trends to the empirical data yields two parameters: κ and ρ. The parameter κ represents the likelihood that a component contains BOLD-related information, whereas ρ represents the likelihood of non-BOLD (noise) contributions. The ratio between these parameters assists in determining whether a component should be retained or rejected (Kundu et al., 2012). Both the optimal combination of the ME time series and the ME-ICA approach individually contribute to an increase in signal-to-noise ratio (SNR) and can be effectively integrated into the same preprocessing pipeline (Steel et al., 2022). Accordingly, some work indicates that ME protocols can yield similar data quality to long SE acquisitions with approximately half the scan length (Lynch et al., 2020). ME-based pipelines can successfully mitigate motion-related contamination but, on their own, do not completely remove the WSDs that are targeted by GSR/GMSR and related techniques (Power et al., 2018).

Several prior studies have compared the relative strengths and weaknesses of some of the common fMRI denoising strategies discussed above. For example, Ciric et al. (2017) compared 14 denoising pipelines for SE data across six metrics, observing considerable between-pipeline variability across various estimates of data quality. The authors concluded that GSR and volume censoring effectively mitigate noise resulting from head motion. Parkes et al. (2018) compared 19 pipelines for SE data. They also found considerable variability in denoising efficacy across pipelines and concluded that pipelines incorporating ICA-AROMA and GSR perform well at mitigating motion-related noise. Few direct comparisons of ME and SE acquisitions have been reported. In one analysis, Dipasquale et al. (2017) compared several popular SE and ME data preprocessing strategies and found that ME-ICA outperformed ICA-AROMA and ICA-FIX according to several established fMRI data quality. RIPTiDe has recently been found to remove sources of noise that more commonly used methods may miss (Korponay et al., 2024).

Most of these studies have used different measures of denoising efficacy or data quality that aim to quantify the level of residual noise (e.g., motion contamination) found in the data after denoising. Although useful, such measures only consider removal of noise and do not quantify preservation of signal of interest. This is important, since a given denoising method may be overly aggressive, removing all noise-related contamination along with neuronal variance. Several alternative approaches have been developed to investigate this question (Aquino et al., 2020; Glasser et al., 2019), but perhaps the simplest strategy involves an evaluation of the degree to which the processing pipeline influences the performance of an FC-based predictive model of some behavioral outcome. Using this approach, Li et al. (2019) showed that SE pipelines incorporating GSR generally yielded models that more accurately predicted distinct aspects of personality and cognition than pipelines omitting this step. Another recent study comparing a wider range of SE pipelines found that no single pipeline consistently excelled at denoising efficacy and behavioral prediction, but that combining ICA-FIX and GSR offered a reasonable balance between these two priorities (Pavlovich et al., 2024).

No study to date has compared different SE and ME data preprocessing strategies across both data quality measures and the ability to predict individual differences in behavior. To this end, we compared 90 different denoising pipelines for single and ME fMRI data, focusing on denoising efficacy and behavioral predictions with respect to seven different measures of personality and cognition. This approach allowed us to investigate whether there is a specific acquisition and processing pipeline that is simultaneously associated with optimal denoising efficacy and behavioral prediction performance.

## METHODS

### Participants

A total of 418 community-dwelling adults aged 18–45 years were recruited for a broader project examining brain–behavior relationships. Inclusion in this analysis was contingent on participants possessing fMRI and T1-weighted images. Participants were recruited via an online campaign targeting various social media and other outlets, coordinated by the private company, Trialfacts (https://trialfacts.com/). Written informed consent was required before study participation. Inclusion criteria for this study included aged 18–45 years; right-handedness; European ancestry (defined as all four grandparents of European descent); no history of frequent headaches or migraines, past seizures, concussions, or loss of consciousness that lasted more than 3 min; English as the first spoken language; normal or corrected-to-normal vision; no metal in the body; no history of neurosurgery; not currently pregnant or attempting to conceive; no history of receiving hormone blockers or hormone replacement; no history of gender-affirming surgery; no history of steroid abuse; no neurological illness; no history of significant head injury inducing loss of consciousness; and no previous experience of electroconvulsive therapy. Experience of psychiatric symptoms or past/current psychiatric diagnosis and/or treatment were also used as a basis for exclusion for the present analysis to minimize sample heterogeneity. Treatment in this context was operationalized as no transcranial magnetic stimulation (TMS) within the past 6 months; no more than 2 weeks of TMS exposure in the last year; no more than 3 months continuous TMS at any point of life; and no history of psychiatric diagnosis, hospitalization, or medication use. All participants provided informed consent in accordance with Monash Human Research Ethics Committee guidelines.

### Data Acquisition

ME-rs-fMRI data were collected on a Siemens 3 T Skyra (Siemens Healthineers, Erlangen, Germany) using an interleaved sequence with the following parameters: isotropic voxel size = 3.2 mm, slices = 40, repetition time (TR) = 0.91 s, phase encoding direction = R-L, echo times TE1 = 12.60 ms, TE2 = 29.23 ms, TE3 = 45.86 ms, TE4 = 62.49 ms, flip angle = 56°, bandwidth = 2,520 Hz/Px, echo spacing = 0.00025, GeneRalized Autocalibrating Partial Parallel Acquisition acceleration factor *R* = 2, volumes = 767, multi-band factor = 4. The total runtime per participant was 12 min. An opposite phase encoding scheme (L-R) with 10 volumes, but otherwise identical parameters, was also acquired for susceptibility distortion correction. T1-weighted images were also acquired using a magnetization-prepared 2 rapid-acquisition gradient-echoes (MP2RAGE) MRI sequence (TR = 5,000 ms, TE = 2.98 ms, TI1/T12 = 700/2,500 ms, 1.0-mm cubic voxel size).

### Data Processing

**Figure 1.**
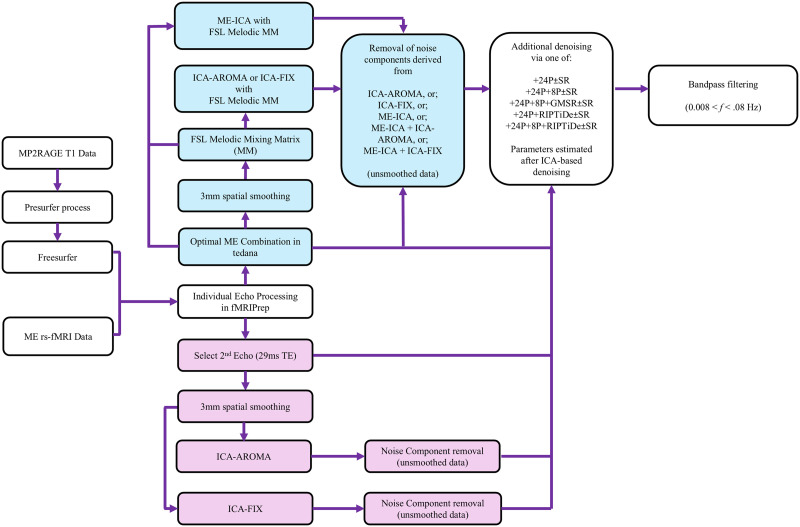
Overview of data preprocessing pipelines. ME-specific preprocessing steps are colored light blue; SE-specific preprocessing steps are colored pink. Common preprocessing steps are in white boxes. MM = mixing matrix; 24P = raw head rotation and translation information, their first-order derivatives, their quadratics, and the quadratics of their derivatives; 8P = average CSF and WM signals, their derivatives, their quadratics, and the quadratics of their derivatives; GMSR = average gray matter signals, their derivatives, their quadratics, and the quadratics of their derivatives; SR = a strategy for volume censoring.

Figure 1 depicts the analysis workflow for our comparative evaluation. The second echo image (i.e., TE = 29.23 ms) was used for all SE preprocessing pipelines. The following provides details of the specific steps applied to the anatomical and functional data.

#### T1-weighted MP2RAGE processing.

The MP2RAGE sequence involves the acquisition of two structural images with different inversion times (INV1 and INV2), which are then combined into a single T1-weighted image (UNI). The combination provides enhanced gray-matter-to-WM contrast and improves tissue segmentation accuracy (Marques et al., 2010; O’Brien et al., 2014) but can increase noise in nonbrain regions, air, and large sinus spaces. To suppress such noise, we generated whole-brain masks and masks of several nonbrain regions from the full INV2 image using the presurfer package (Kashyap et al., 2021; https://github.com/srikash/presurfer). These masks were then used to strip regions affected by noise in the spatially aligned UNI image. Visual quality control confirmed the success of this approach. The processed full-head UNI image was then used as input to the Freesurfer recon-all pipeline for denoising, cortical reconstruction, and segmentation (Dale et al., 1999; https://surfer.nmr.mgh.harvard.edu/). The resulting images were then incorporated into the fMRI processing pipelines, as detailed below.

#### Functional data processing—minimal preprocessing (MP).

Functional MRI images were minimally preprocessed using the container fMRIPrep 23.1.3 (Esteban et al., 2019), based on Nipype 1.8.6 (Gorgolewski et al., 2011; https://fmriprep.org/en/stable/). In brief, for anatomical data, this involved the creation of spatial maps quantifying the voxelwise probability of gray matter, WM, or CSF (Zhang et al., 2001), followed by registration to standard space (MNI152NLin2009cAsym; Avants et al., 2008). Freesurfer masks and surfaces were used to refine brain masks derived via antsBrainExtraction (Tustison et al., 2021) per fMRIPrep-specific sequences (i.e., from aseg.mgz). fMRI data were corrected for head motion via rigid body alignment (Jenkinson et al., 2002) and for susceptibility distortions using the reverse polarity image (Andersson et al., 2003; Smith et al., 2004). The functional scans were then co-registered to T1w space using boundary-based registration (Greve & Fischl, 2009) and spatially normalized to MNI152NLin2009cAsym space using nonlinear registration (Avants et al., 2008). These same steps were applied independently to the fMRI time series obtained at each echo time, but parameters required for head motion correction and susceptibility distortion correction were estimated from the first echo and then applied to the remaining echo data, as per the default procedure in fMRIPrep. Slice timing correction was not performed due to the short TR of the fMRI acquisition sequence.

#### ME optimum combination (OC).

A T2* map was estimated from the individually preprocessed echoes by fitting the maximal number of echoes with reliable signal for a given voxel to a monoexponential signal decay model using nonlinear regression, and the calculated T2* map was then used to optimally combine preprocessed BOLD data across echoes, following Posse et al. (1999). Specifically, data across echoes were combined using a weighted average that prioritizes information from echo times near 30 ms for superior signal dropout to tissue contrast ratios. This step was executed using the software package *tedana* (DuPre et al., 2021; https://tedana.readthedocs.io/en/stable/).

#### FMRIB Software Library (FSL) Multivariate Exploratory Linear Optimized Decomposition into Independent Components (MELODIC).

For SE pipelines, s-ICA was run using the FSL-MELODIC tool, performed on a 3-mm spatially smoothed preprocessed second echo image (https://web.mit.edu/fsl_v5.0.10/fsl/doc/wiki/MELODIC.html).

For ME pipelines, the preprocessed, optimally combined BOLD images were smoothed using a 3-mm Gaussian kernel and then subjected to s-ICA with FSL-MELODIC (Beckmann & Smith, 2004), yielding a mixing matrix in which each column represents an estimated IC and rows represent volumes. Using this mixing matrix as input for all subsequent ICA-based denoising strategies ensured that all algorithms were parsing signal and noise using the same basis.

#### ICA-AROMA.

The corresponding FSL-MELODIC outputs for SE and ME pipelines were used as input for ICA-AROMA (Pruim et al., 2015; https://github.com/maartenmennes/ICA-AROMA). The algorithm was run using the reference BOLD image (a static image created via the median of motion-corrected volumes) created by fMRIPrep. The preprocessed T1w image created by fMRIPrep was skull-extracted using the FSL-Brain Extraction Tool (https://web.mit.edu/fsl_v5.0.10/fsl/doc/wiki/BET(2f)UserGuide.html). The resulting brain mask and skull-extracted images were also supplied to MELODIC. All images were spatially aligned using boundary-based registration (Greve & Fischl, 2009).

#### ME-ICA.

The nonsmoothed individually preprocessed echoes were processed with *tedana* for optimal combination and ME-ICA. ME-ICA applies automatic rejection of components based on the associated *κ*/*ρ* ratio. These terms reflect the degree of model fit for known BOLD and non-BOLD T_2_* trends with respect to the empirical data (Kundu et al., 2012). The MELODIC-estimated mixing matrix was used as input for the algorithm to ensure equivalence between pipelines. No smoothing was applied, as is standard for ME-ICA (Kundu et al., 2012).

#### ICA-FIX.

ICA-FIX involves hand classification of ICs estimated by FSL-MELODIC as either “signal” or “noise” within a training set derived from a larger collection of data. Hand-labeling of noise components was conducted twice for the same 20 subjects (10 male, 10 female) after FSL-MELODIC, once for their smoothed SE data and once for their smoothed optimally combined ME data. As such, separate training sets were generated for the SE and ME processing streams.

Manual classification was performed according to robust heuristics previously explicated in the literature (Griffanti et al., 2017). Leave-one-out cross-validation was used to assess classification accuracy, which involved iteratively training the classifier on all but one participant and using the left-out participant to evaluate the classifier’s alignment with manual classifications. This alignment was assessed over a variety of rejection thresholds, which determine how aggressive the algorithm is in identifying noise components. Subject-wise true positive rates (TPRs) and true negative rates (TNRs) were averaged across the 20 participants to estimate overall classifier performance for each rejection threshold. Previous literature suggests that TPR should approximate 90%, while TNR can be as low as 10%, to maintain the maximum amount of signal (Carone et al., 2017). For both our SE and ME datasets, a rejection threshold of 40 was identified as optimal for balancing TPR and TNR (SE: TPR [mean, median] = [87.70%, 88.10%]; TNR [mean, median] = [86.40%, 87.80%]; ME: TPR [mean, median] = [91.10%, 91.40%]; TNR [mean, median] = [89.00%, 89.90%]).

#### Removing ICA-derived noise signals.

For ME pipelines combining ICA-based denoising strategies (e.g., ME-ICA + ICA-AROMA), the list of noise components extracted via each denoising algorithm was combined into a single vector describing the noise component index positions (duplicates removed). The mixing matrix and the full list of rejected components was then supplied to FSL’s fsl_regfilt function for denoising via nonaggressive ordinary least squares regression.

#### 8P, GMSR, and spike regression (SR).

Classical 8P, 8P + GMSR, and SR were applied in a second step, after ICA-based denoising, according to established analysis guidelines (Aquino et al., 2022; Parkes et al., 2018; Power, 2017). Probabilistic masks for Grey Matter (GM), WM, and CSF estimated from anatomical data during fMRIPrep were spatially aligned to the BOLD image (Avants et al., 2008). To minimize partial volume effects (e.g., GM inclusion in the WM mask), the CSF mask was eroded once using a 3 × 3 × 3 mm kernel, whereas the WM mask was eroded up to five times. If an erosion iteration left fewer than five voxels in the mask, the previous erosion iteration was used. Mean time-series data within each of these eroded masks were then extracted from the preprocessed data (i.e., after any ICA-denoising, if applied). The first derivatives, their powers, and the powers of the raw time-series data were also calculated individually for the WM, CSF, and gray matter tissue areas. These were then concatenated with the 24P motion parameters calculated in fMRIPrep to create participant-specific confound matrices. GMSR was performed with the associated expansion and derivative terms (four parameters total).

We also ran all of our pipelines with and without SR as a censoring strategy. Frames targeted for censoring were identified with respect to participant-specific FD measures (Yan et al., 2013). FD quantifies degree of head motion and is estimated from head translation and rotation. To account for pseudomotion arising due to the rapid TR of the multiband sequence, we followed prior work (Fair et al., 2020) and filtered the six rotation and translation head motion parameters using a band-stop Butterworth filter (0.31–0.41 Hz). Summed absolute successive differences were computed from these processed vectors and used to re-estimate FD for each time point (Fair et al., 2020). Timepoints exceeding 0.20 mm in FD were flagged for SR. If fewer than 5 timepoints occurred between periods flagged for SR, these originally unmarked volumes were also included for SR (Gordon et al., 2016). 8P regression ± GMSR (with and without SR) were performed in a single step and subsequent to any ICA-based denoising using the regression model implemented in fsl_regfilt (Lindquist et al., 2019; Satterthwaite et al., 2013).

#### RIPTiDe.

The toolbox Rapidtide was used to estimate LFOs via the RIPTiDe algorithm (Tong et al., 2019; https://github.com/bbfrederick/rapidtide). Time delay analyses were performed for each gray-matter voxel’s time series to assess similarity with a reference time series in the LFO band. More specifically, a “probe regressor” was derived from empirical data by filtering the average gray matter BOLD signal to frequencies within the LFO band (i.e., 0.01 Hz < *f* < 0.15 Hz) to capture hemodynamic fluctuations. Time-lagged voxelwise BOLD amplitude changes were then cross-correlated with this probe regressor to identify the most strongly correlated lag within a time window of ±10 s, with probe-related (i.e., blood arrival related) variance then removed from the voxel time series using linear regression. This step was performed as an alternative to GMSR, prior to bandpass filtering. Pipelines involving 24P and ICA-AROMA can help improve LFO estimation by reducing motion artifacts without removing hemodynamic noise, but RIPTiDe is not validated for data denoised with 8P, ICA-FIX, or ME-ICA, which aim to mitigate sources of noise that may overlap with those targeted by RIPTiDe. In pipelines involving 8P, ICA-FIX, or ME-ICA, RIPTiDe regressors were estimated from the data with only 24P applied. The RIPTiDe regressors were then removed from the data in a separate stage to which 8P, ICA-FIX, or ME-ICA regressors were applied, as is the default.

#### FC estimation.

The gray matter probability map derived from fMRIPrep and the preprocessed BOLD images were registered to MNI152NLin2009cAsym space (Avants et al., 2008) and multiplied together to limit partial volume effects and assign higher weight to gray matter voxels when extracting regional mean time series (see below). The resulting functional data were bandpass filtered (0.008 < *f* < .08 Hz) before parcellation using the combined Schaefer400 and Melbourne Scale 2 Subcortical Atlas, dividing the cortex into 400 and the subcortex into 32 functionally homogenous regions (Schaefer et al., 2018; Tian et al., 2020).

The mean time series for each region was extracted and individual-specific FC matrices were calculated using product–moment correlations between regional time courses, as implemented in Nilearn, based on Nipy (Brett et al., 2020; Gorgolewski et al., 2011). These correlation values were then transformed into *z*-scores using Fisher’s *r*-to-*z* transformation (Fisher, 1915).

#### Quality control metrics (QCMs).

Six data quality evaluation metrics were assessed for each pipeline: (a) variance explained by the first principal component of the parcellated time series (VE1); (b) delta variation signal (DVARS); (c) temporal signal-to-noise ratio (TSNR); (d) quality control functional connectivity (QC-FC); (e) QC-FC-distance dependence; and (f) FCI.

VE1 characterizes the extent to which the FC estimates are globally correlated. Higher values suggest strong WSD contamination, potentially arising from major fluctuations in respiration and/or head motion (Aquino et al., 2020). DVARS measures the similarity of voxel signal amplitude between consecutive time points (Afyouni & Nichols, 2018; Power et al., 2012). Averaging DVARS across the brain mask provides an overall metric of voxel signal homogeneity over time. High values indicate strong fluctuations in signal intensity over time, which may be induced by head motion. TSNR is estimated as the mean signal of a voxel over time divided by its standard deviation. Unlike DVARS, higher average TSNR values across the brain indicate greater stability, while lower values suggest more variability, which may be driven by noise (Murphy et al., 2007).

QC-FC is estimated at each edge and quantifies the degree to which a given FC estimate correlates with a summary measure of head motion, quantified using mean FD, across participants (Aquino et al., 2020; Ciric et al., 2017; Parkes et al., 2018). The distribution of QC-FC values should have a mode of zero and minimal variance following the successful mitigation of motion-related artifacts. QC-FC distance dependence reflects the degree to which QC-FC relationships correlate with the Euclidean distances between each pair of regions. High head motion can drive higher FC estimates for short-range connections and lower FC for long-range connections (Ciric et al., 2017; Power et al., 2012).

Finally, FCI refers to an artifactual increase of FC as a function of time spent in the scanner (Korponay et al., 2024). Many contemporary preprocessing strategies are incapable of effectively removing this artifact (Korponay et al., 2023). Twenty consecutive windows (approximately 34 s each) were investigated for the present analyses. Previous research on FCI has used time-windows as short as 21.6 s (Korponay et al., 2023), but larger window sizes (approaching 40 s) may allow for greater stability in estimates of dynamic changes in FC (Zalesky & Breakspear, 2015; Zhuang et al., 2020).

#### Participant exclusion.

Sixty participants were removed on the basis functional data preprocessing failures, session interruption or incomplete fMRI data (i.e., fewer than 767 volumes collected), mean FD exceeding 0.25 mm, > 20% of FDs exceeding 0.20 mm, any FDs exceeding 5.00 mm, or if > 50% of their data were flagged for SR (Aquino et al., 2020; Parkes et al., 2018; Pavlovich et al., 2024; final *n* = 358).

### Behavioral Measures and Predictions

The 60-item Big 5 Inventory II measures the Big Five Personality Traits: Neuroticism, Extraversion, Conscientiousness, Agreeableness, and Openness. Participants respond on 5-point Likert scales from 1 (*disagree strongly*) to 5 (*agree strongly*; Soto & John, 2017). Intelligence was also measured via the Wechsler Abbreviated Scale of Intelligence–Second Edition (WASI-II; Wechsler, 1999, 2018). More specifically, we measured Vocabulary and Matrix Reasoning scores.

After exclusions, 4.64% of survey data were missing across the 358 retained participants and variables. Missing data in behavioral measures were imputed using predictive mean matching, a widely used statistical method generally associated with greater accuracy in missing data estimation relative to other imputation methods (Austin et al., 2021; Bailey et al., 2020; Morris et al., 2014). Imputation was conducted using the multivariate imputation by chained equations (MICE) approach implemented in the *mice* package (van Buuren & Groothuis-Oudshoorn, 2011) written for R (R Core Team, 2013). Although this approach typically generates multiple datasets with different imputations for missing values, a single imputed dataset was selected at random for input into subsequent Kernel Ridge Regression (KRR) analyses to provide an almost unbiased estimate of the missing data.

## RESULTS

### Evaluation of Pipeline-Level QCMs

**Figure 2.**
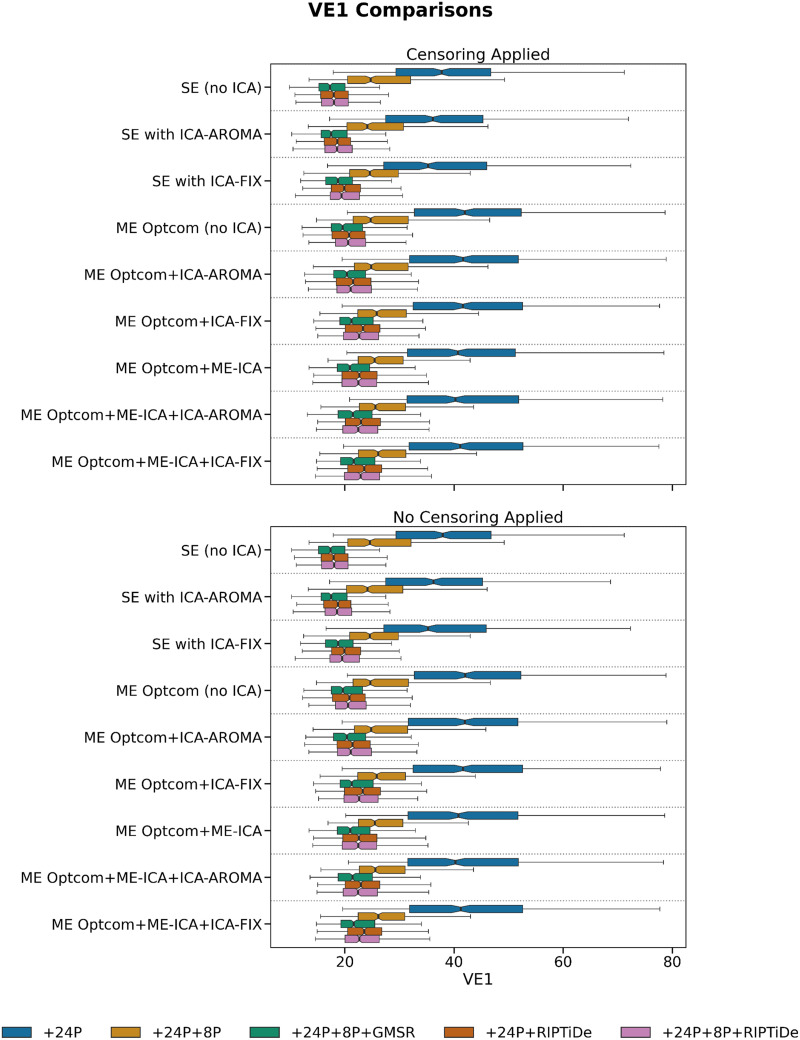
The effect of various denoising strategies on subject-level VE1, aggregated at the pipeline level. Smaller values indicate less noise. The best performing pipelines were those in which GMSR or RIPTiDe were applied, regardless of ICA type.

**Figure 3.**
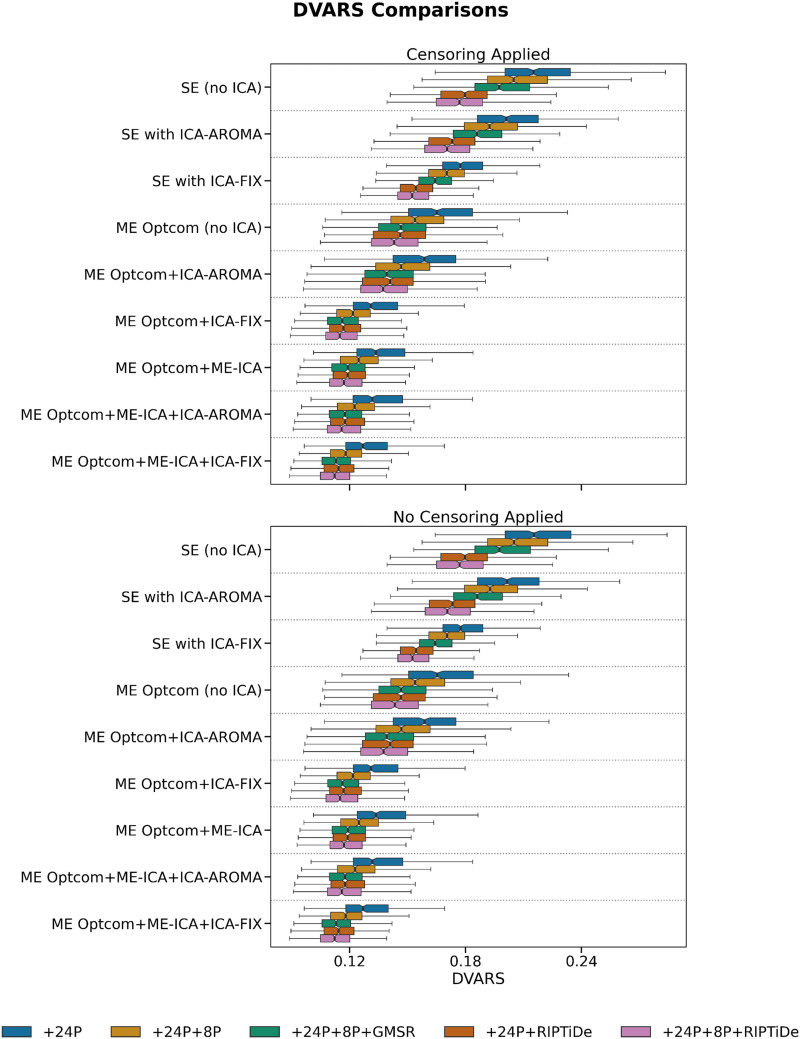
The effect of various denoising strategies on subject-level DVARS, aggregated at the pipeline level. Smaller values indicate less noise. The best performing pipelines were generally those in which more preprocessing steps were applied.

**Figure 4.**
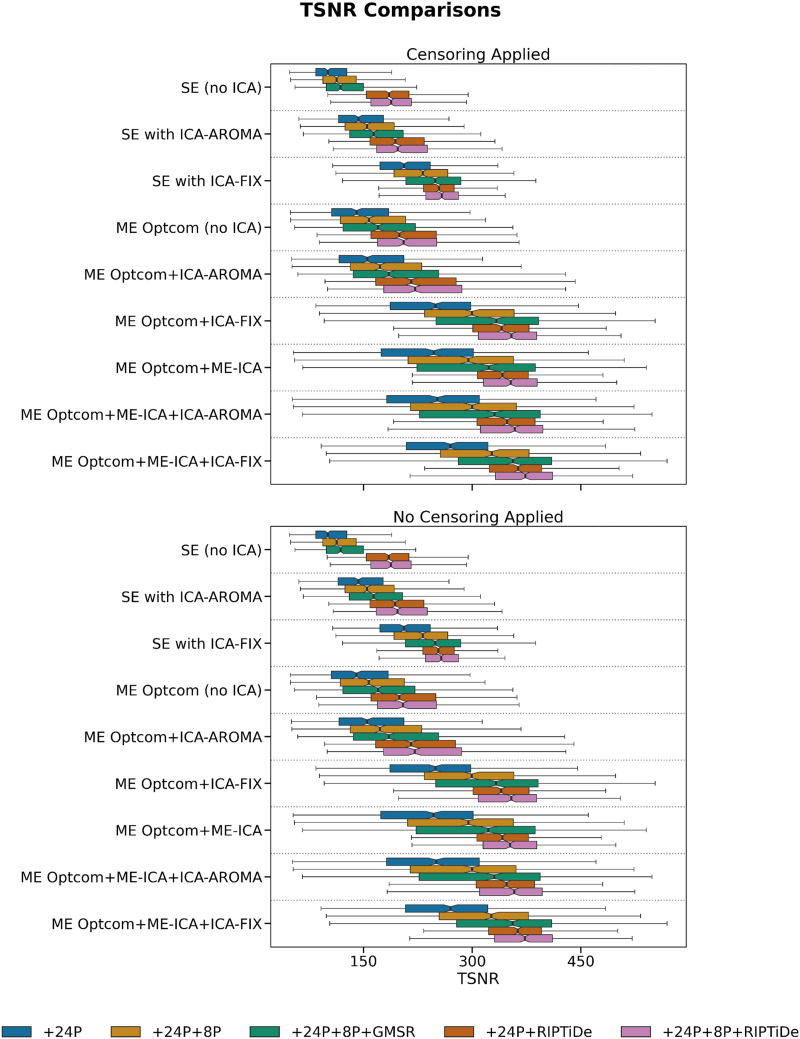
The effect of various denoising strategies on subject-level TSNR, aggregated at the pipeline level. Larger values indicate less noise. The best performing pipelines were generally those in which more preprocessing steps were applied.

We first evaluated six QCMs per pipeline: VE1, DVARS, TSNR, QC-FC, QC-FC Distance, and FCI. As expected, the removal of WSDs via either GMSR or RIPTiDE led to a dramatic reduction of VE1, with no obvious differences between SE and ME data or between different ICA denoising strategies (Figure 2). For DVARS and TSNR, ME pipelines showed a major advantage over SE pipelines. Applying ME-ICA with GMSR or RIPTiDE led to further improvements on these benchmarks. There was a marginal gain offered by combining ICA-FIX with ME-ICA compared with combining ME-ICA with ICA-AROMA (Figures 3 and 4). For SE data, ICA-based pipelines led to better DVARS and TSNR estimates compared with non-ICA-based pipelines, with ICA-FIX performing better than ICA-AROMA.

**Figure 5.**
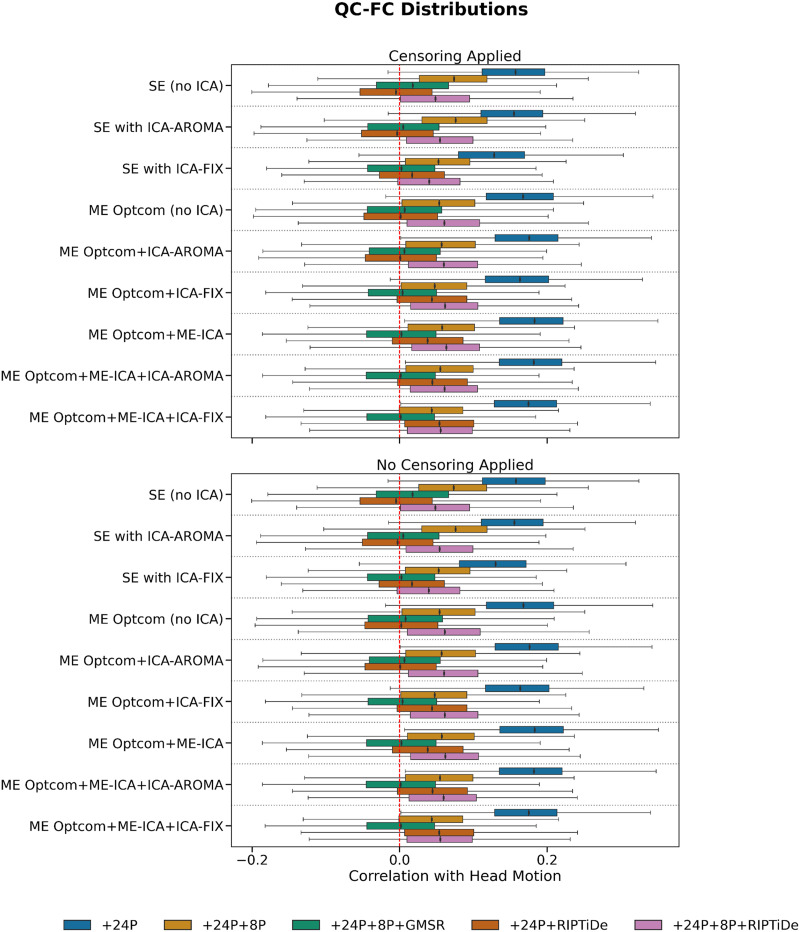
The effect of various denoising strategies on subject-level QC-FC, aggregated at the pipeline level. Correlations are Spearman’s rho (ρ). Distributions centered at zero indicate less head motion contamination. Pipelines with GMSR or RIPTiDe generally performed best regardless of ICA type.

**Figure 6.**
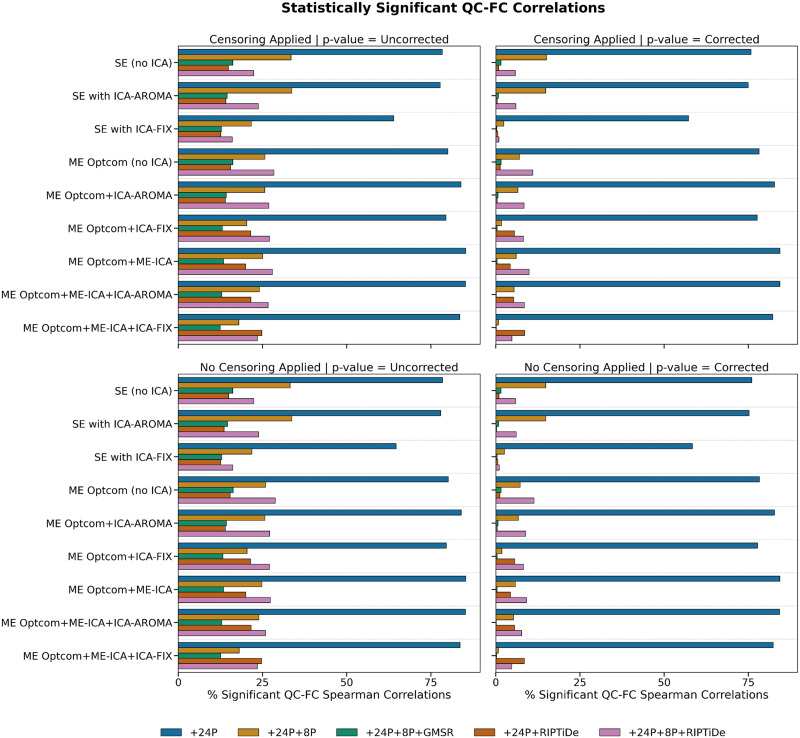
The effect of various denoising strategies on the number of statistically significant QC-FC correlation values (uncorrected values displayed left columns; Benjamini–Hochberg-corrected *p* values right columns; censoring applied in top row; no censoring applied in bottom row). A larger overall percentage of edges significantly correlated with head motion indicates greater amounts of head motion contamination in the data. Pipelines with GMSR or RIPTiDe and 24 head parameter regression generally performed best regardless of ICA type.

**Figure 7.**
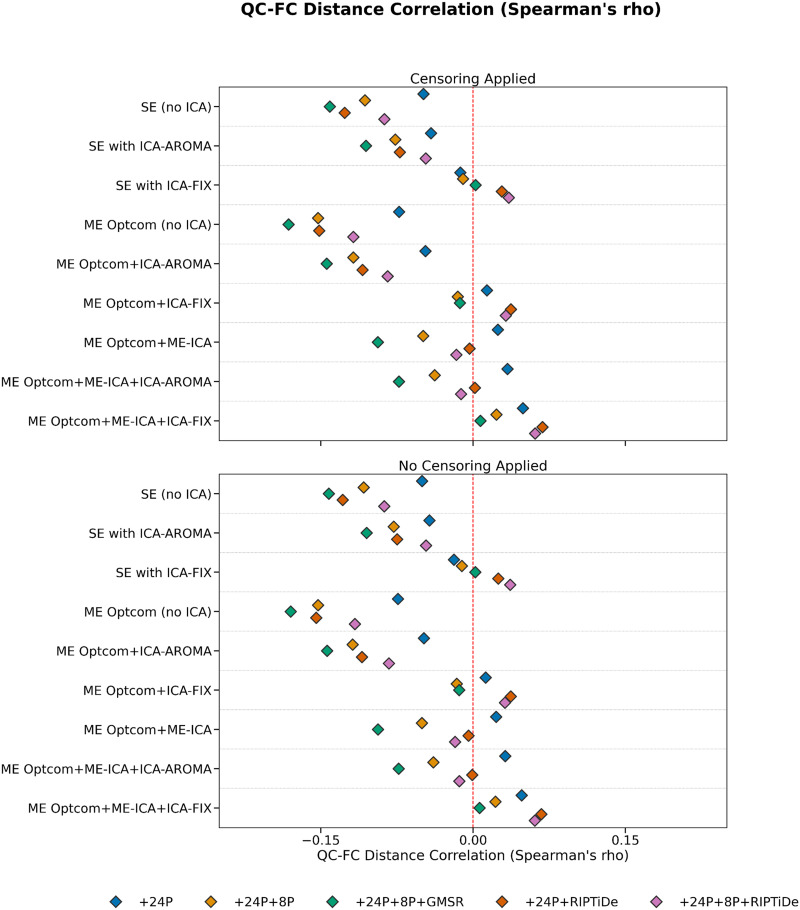
The effect of various denoising strategies on pipeline-level QC-FC distance dependence. Correlations are Spearman’s rho (ρ). Distributions centered at zero indicate less head motion contamination in the data. In SE data, the best-performing pipelines for reducing distance dependence used ICA-FIX. In ME data, the application of ME-ICA generally improved distance dependence relative to pipelines that did not use ME-ICA, with the best-performing ME pipelines combining ME-ICA, ICA-FIX, and GMSR, or combining ME-ICA, ICA-AROMA, and GMSR.

**Figure 8.**
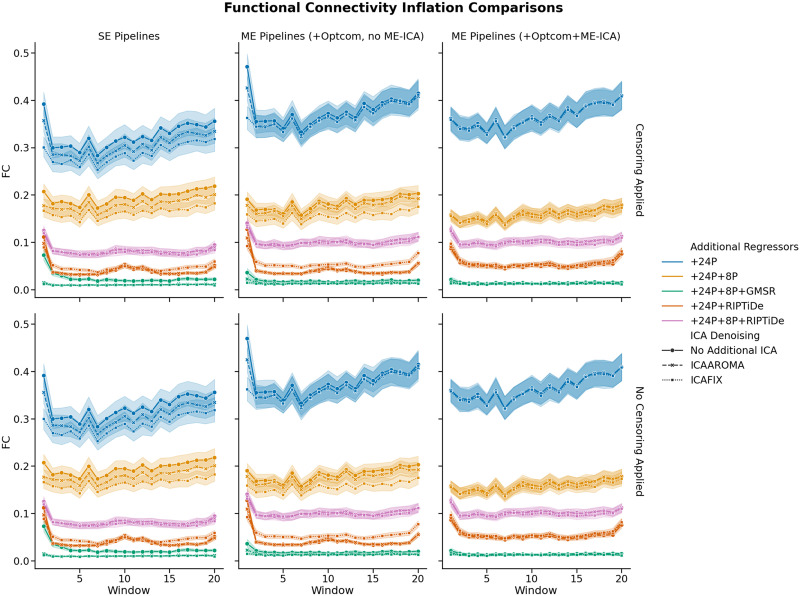
The effect of various denoising strategies on subject-wise FCI for single and ME data, aggregated at the pipeline level. Censoring was applied for pipelines displayed in the top row. No censoring was applied for pipelines in the bottom row. Individual time series were discretized into 20 windows. Lack of line slope indicates diminished FCI. No trend was observed for censoring status, or whether SE data were used or whether OC or ME-ICA was applied. Note that additional ICA denoising categories overlap considerably and that differences only emerged based on the nature of the additional regressor during the secondary denoising stage.

Pipelines with some form of WSD removal (i.e., GMSR or RIPTiDE) improved QC-FC correlation (*ρ*) distributions (i.e., the mode of the distribution was closer to zero; Figure 5) and reduced the number of edges demonstrating significant relationships (*p* < .05, uncorrected) with head motion (Figure 6). We also observed a consistent, yet minor, gain for GMSR over RIPTiDE across SE and ME pipelines for these metrics. QC-FC distance dependence was more variable overall, ranging between *ρ* = −.18 (for ME:MP + OC + 24P + 8P + GMSR) and *ρ* = .07 (for ME:MP + OC + MEICA + ICAFIX + 24P + RIPTiDe; Figure 7). In SE data, the best-performing pipelines for reducing distance dependence used ICA-FIX. In ME data, the application of ME-ICA generally improved distance dependence relative to pipelines that did not use ME-ICA, with the best-performing ME pipelines combining ME-ICA, ICA-FIX, and GMSR, or combining ME-ICA, ICA-AROMA, and GMSR. The application of RIPTiDE instead of GMSR in these pipelines led to a slight worsening of QC-FC distance dependence (e.g., from *ρ* = −.01 for pipeline ME:MP + OC + MEICA + ICAAROMA + 24P + 8P + RIPTiDe to *ρ* = .07 for pipeline ME:MP + OC + MEICA + ICAFIX + 24P + RIPTiDe). FCI was evident in both SE and ME data and was generally only mitigated when some form of WSD removal (GMSR or RIPTiDE) was used (Figure 8). Censoring was not associated with major variations in any of the data quality metrics. For each plot, pipelines are presented in order of increasing denoising complexity, where complexity was approximated by the number of additional preprocessing operations implemented in each pipeline.

### Behavioral Predictions

We next evaluated behavioral prediction accuracies using cross-validated FC-based KRR (J. Li et al., 2019). Aggregating results across both cognitive and personality measures via averaging (Figure 11), the best performance was achieved for ME pipelines that did not use WSD removal. Specifically, four ME pipelines stood out as having the highest accuracies: ME:MP + OC + ICA-FIX + 24P, ME:MP + OC + ME-ICA + 24P, ME:MP + OC + ME-ICA + ICA-FIX + 24P, and ME:MP + OC + ME-ICA + ICA-AROMA + 24P. Of ME pipelines including WSD removal, pipelines combining ICA-AROMA without ME-ICA showed higher average performance than those using ME-ICA or ICA-FIX. There was a slight performance disadvantage for pipelines combining ME-ICA, ICA-FIX, and WSD removal, suggesting that these pipelines may be overly aggressive and remove behaviorally relevant variance despite their advantages on QCMs.

**Figure 9.**
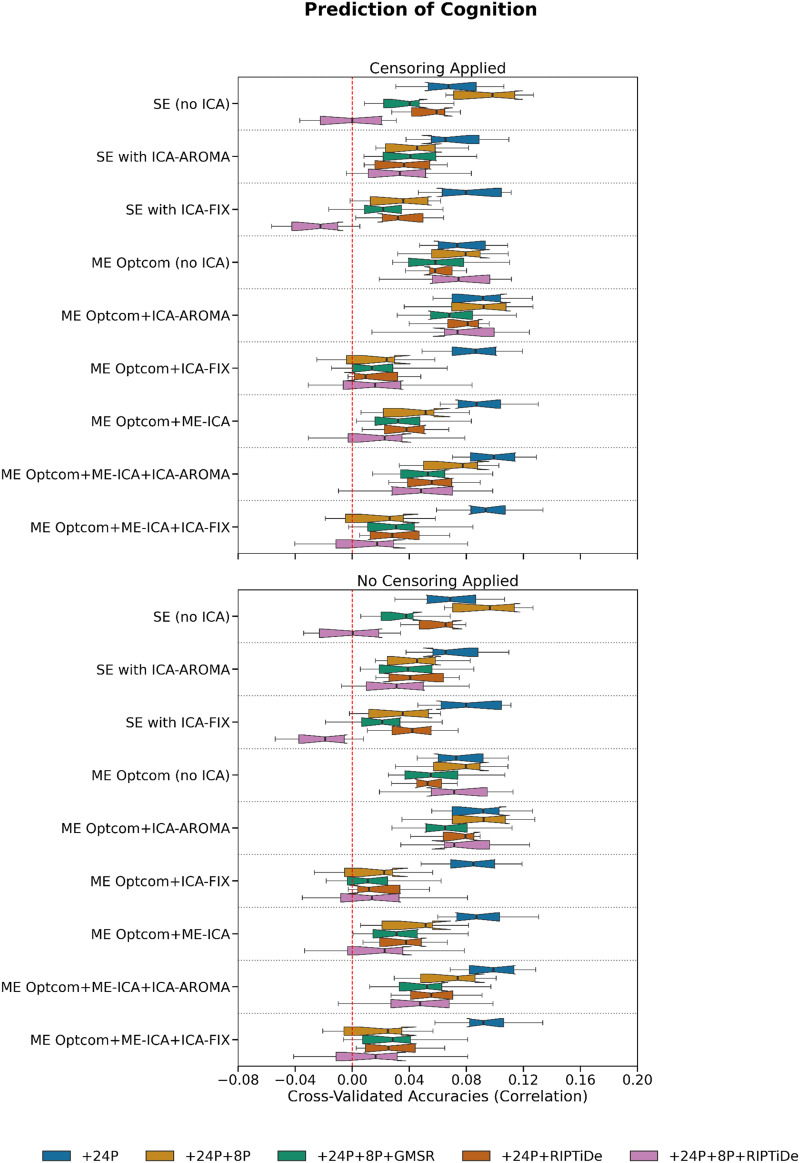
The effect of various denoising strategies on pipeline-level cognition prediction. Cognitive measures were individual-level *T*-score transformed WASI-II Matrix Reasoning and WASI-II Vocabulary scores, averaged across the two tasks. The results reported here are typical of machine learning neuroimaging studies.

**Figure 10.**
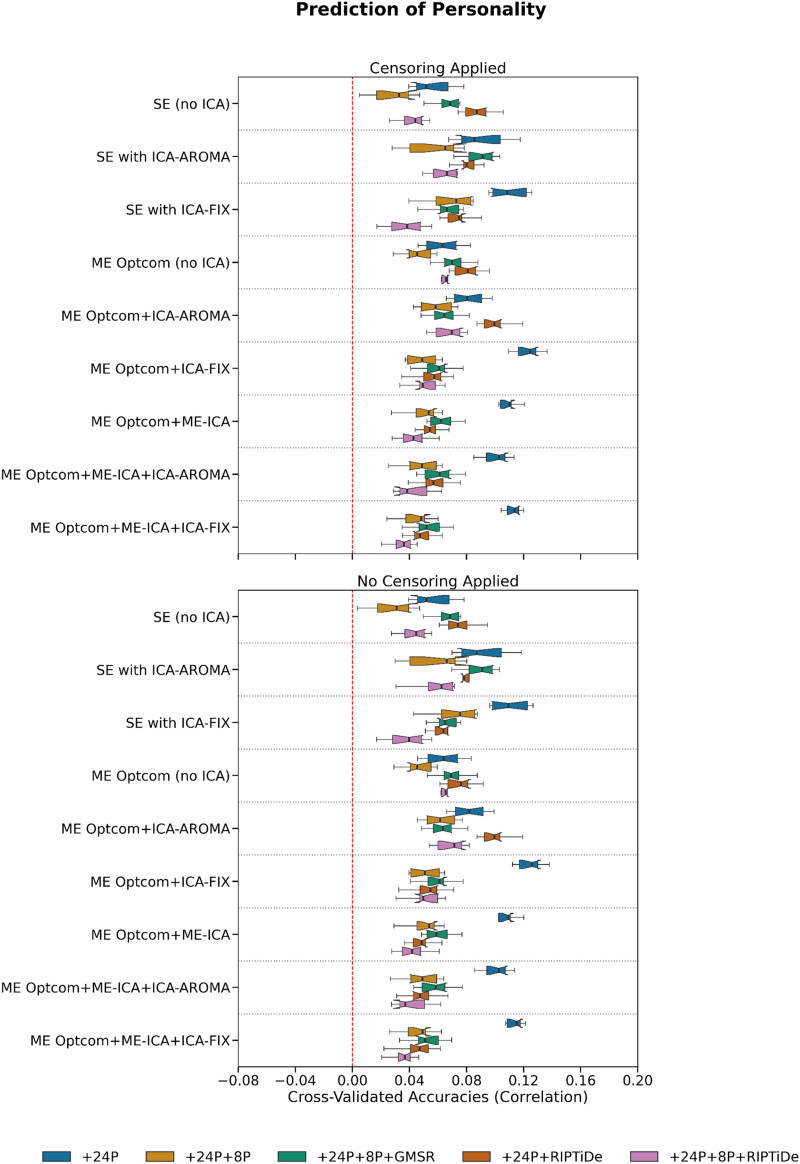
The effect of various denoising strategies on pipeline-level personality prediction. Personality measures were individual-level Big Five (domain) scores. The results reported here are typical of machine learning neuroimaging studies.

**Figure 11.**
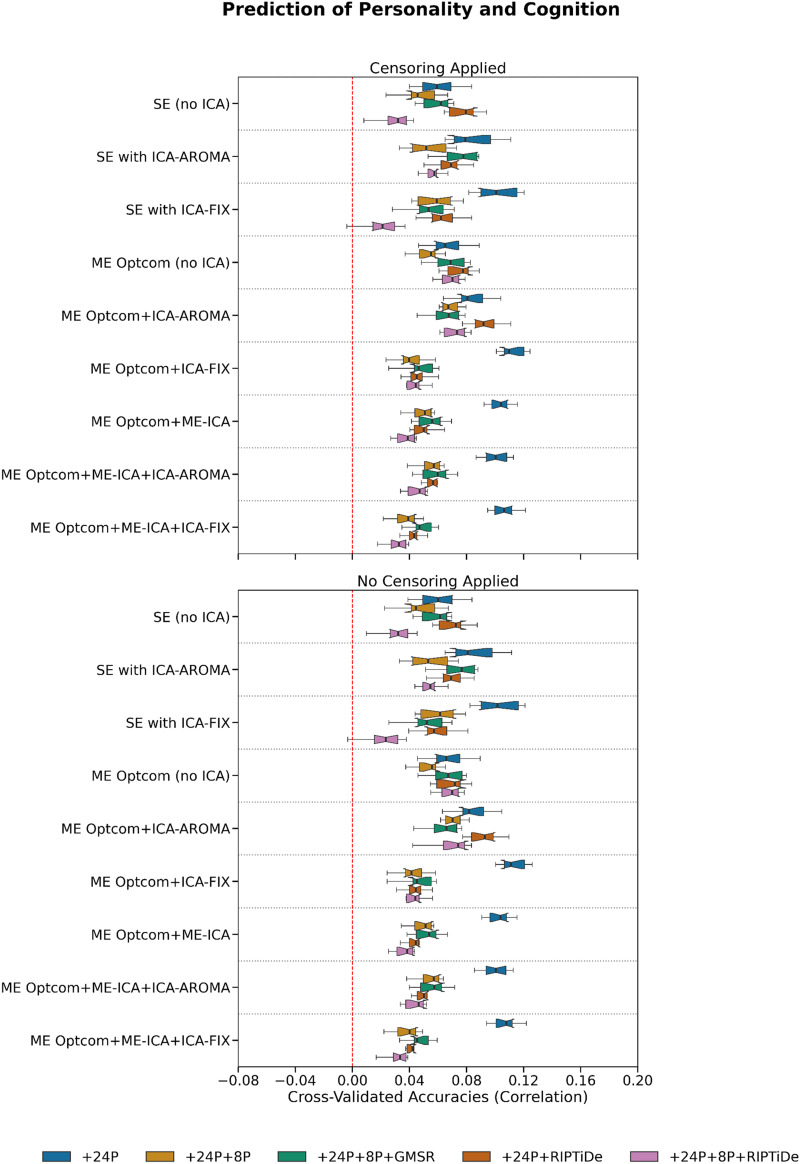
The effect of various denoising strategies on pipeline-level personality and cognition prediction. Personality measures were individual-level Big Five (broad domain) scores, and cognitive measures were individual-level *T*-score transformed WASI-II Matrix Reasoning and WASI-II Vocabulary scores. The results reported here are typical of machine learning neuroimaging studies.

Of the pipelines that were not ranked in the top 4, SE pipelines generally showed higher average performance than ME pipelines, with some evidence that WSD removal led to decreased predictive accuracy. Similar trends were observed when inspecting results for cognition and personality separately (Figures 8 and 9). Censoring had minimal effect on model accuracies. Note that while performance differences between some pipelines exceeded 100%, prediction accuracies were generally small and never exceeded *r* = 0.15 (Figures 9, 10, and 11).

### Pipeline Rankings

We ranked each pipeline based on its performance across multiple metrics using two methods: ordinal rankings and percentage decrement. In the ordinal ranking scheme, we assigned a rank of 1 to the top-performing pipeline and progressively ranked other pipelines in order with higher numbers. As such, lower values indicated better performance. Since it is difficult to quantify and rank pipelines in their degree of FCI improvement, we simply assigned a score of 1 to pipelines that removed FCI (i.e., pipelines using RIPTiDe or GMSR) and a score of 2 to all others. In the percentage decrement ranking scheme, the best performing pipeline was assigned a score of 100% and all other pipelines were ranked as a percentage decrement relative to this value (with lower percentages indicating worse performance). For FCI, pipelines incorporating the RIPTiDe algorithm or GMSR were assigned a score of 100% and all others received a score of 0%. To obtain an overall rank for each pipeline’s denoising efficacy, we averaged the rankings across denoising metrics from both the ordinal and percentage decrement schemes independently. The same approach was applied to KRR results (i.e., averaging ranks across Figures 9 and 10 independently before combination), yielding overall ranks for behavioral predictions. These ranks were then averaged to obtain an overall performance ranking for each pipeline. Note that these rankings weight QC and behavioral performance benchmark metrics equally and should thus be considered as heuristics, as different investigators may wish to prioritize specific benchmarks in certain studies. The top noncensored ME and SE rankings (according to ordinal rankings) are shown in Table 1; full results can be found in Supporting Information 1. The rankings were largely the same when censoring was incorporated into the pipeline. ME optimally combined images with ICA-AROMA, 24 head motion parameter regression, and RIPTiDe, performed the best overall. Excluding the binary ranking associated with FCI removal did not change the finding.

**Table 1.** Top 10 (noncensored) ME and SE pipelines with regard to the overall performance composite, displayed in ascending order (according to ordinal rankings)

	Pipeline	Overall ordinal rank	Overall percentage decrement
Top 10 ME pipelines	ME:MP + OC + ICAAROMA + 24P + RIPTiDe	24.39	75.90%
	ME:MP + OC + ICAFIX + 24P	27.89	63.49%
	ME:MP + OC + MEICA + ICAFIX + 24P	28.54	61.20%
	ME:MP + OC + MEICA + ICAAROMA + 24P	29.29	59.18%
	ME:MP + OC + MEICA + 24P	30.64	58.84%
	ME:MP + OC + MEICA + ICAAROMA + 24P + 8P + GMSR	34.50	64.22%
	ME:MP + OC + ICAAROMA + 24P + 8P + RIPTiDe	34.93	67.22%
	ME:MP + OC + 24P + RIPTiDe	35.96	61.56%
	ME:MP + OC + ICAAROMA + 24P + 8P + GMSR	37.25	62.09%
	ME:MP + OC + MEICA + ICAFIX + 24P + 8P + GMSR	37.68	61.16%
Top 10 SE pipelines	SE:MP + ICAFIX + 24P	32.25	59.52%
	SE:MP + ICAFIX + 24P + RIPTiDe	33.04	62.47%
	SE:MP + ICAAROMA + 24P + RIPTiDe	33.82	62.26%
	SE:MP + 24P + RIPTiDe	35.25	62.15%
	SE:MP + ICAAROMA + 24P + 8P + GMSR	36.93	60.90%
	SE:MP + ICAFIX+24P + 8P + GMSR	37.39	58.06%
	SE:MP + ICAAROMA + 24P	41.04	44.53%
	SE:MP + ICAFIX + 24P + 8P	41.75	50.74%
	SE:MP + 24P + 8P + GMSR	44.86	50.39%
	SE:MP + ICAAROMA +24P + 8P + RIPTiDe	47.96	54.17%

This was computed via the average of overall denoising efficacy and overall behavioral prediction rank. Censoring did not appear to make a difference with regard to pipeline performance at this coarse level of measurement. MP refers to minimal preprocessing with fMRIPrep.

We also present top performing pipelines for denoising efficacy alone and behavioral prediction alone in Tables 2 and 3, respectively. The pipeline using ME optimally combined images with chained ME-ICA and ICAFIX plus 24 head motion parameter, CSF, WM, and Grey Matter Signal (GMS) regression performed best when only considering denoising efficacy metrics. The ME optimal combination with ICAFIX plus 24 head motion parameter regression performed the best when only considering behavioral prediction results.

**Table 2.** Top 10 (noncensored) ME and SE pipelines with regard to average denoising efficacy performance, displayed in ascending order (according to ordinal rankings)

	Pipeline	Overall ordinal rank	Overall percentage decrement
Top 10 ME pipelines	ME:MP + OC + MEICA + ICAFIX + 24P + 8P + GMSR	9.86	95%
	ME:MP + OC + ICAFIX + 24P + 8P + GMSR	14.86	93%
	ME:MP + OC + MEICA + ICAAROMA + 24P + 8P + GMSR	21.00	87%
	ME:MP + OC + MEICA + 24P + RIPTiDe	23.14	92%
	ME:MP + OC + MEICA + ICAAROMA + 24P + RIPTiDe	23.71	92%
	ME:MP + OC + MEICA + 24P + 8P + GMSR	23.86	85%
	ME:MP + OC + MEICA + ICAFIX + 24P + 8P	26.86	73%
	ME:MP + OC + MEICA + ICAFIX + 24P + 8P + RIPTiDe	27.29	89%
	ME:MP + OC + MEICA + ICAAROMA + 24P + 8P + RIPTiDe	28.00	90%
	ME:MP + OC + ICAFIX + 24P + RIPTiDe	28.29	89%
Top 10 SE pipelines	SE:MP + ICAFIX + 24P + 8P + GMSR	20.29	85%
	SE:MP + ICAFIX + 24P + RIPTiDe	22.57	85%
	SE:MP + ICAFIX + 24P + 8P + RIPTiDe	28.14	83%
	SE:MP + ICAAROMA + 24P + RIPTiDe	32.14	76%
	SE:MP + ICAFIX + 24P + 8P	38.00	62%
	SE:MP + ICAAROMA + 24P + 8P + GMSR	38.86	70%
	SE:MP + 24P + RIPTiDe	40.00	70%
	SE:MP + ICAAROMA + 24P + 8P + RIPTiDe	40.43	76%
	SE:MP + 24P + 8P + RIPTiDe	44.00	71%
	SE:MP + 24P + 8P + GMSR	44.71	63%

This was computed via the average of all denoising metric results. Censoring did not appear to make a substantial difference with regards to pipeline performance at this coarse level of measurement. MP refers to minimal preprocessing with fMRIPrep.

**Table 3.** Top 10 (noncensored) ME and SE pipelines with regard to average behavioral prediction performance, displayed in ascending order (according to ordinal rankings)

	Pipeline	Overall ordinal rank	Overall percentage decrement
Top 10 ME pipelines	ME:MP + OC + MEICA + ICAFIX + 24P	4.50	90%
	ME:MP + OC + MEICA + ICAAROMA + 24P	6.00	88%
	ME:MP + OC + ICAFIX + 24P	7.50	92%
	ME:MP + OC + MEICA + 24P	8.00	87%
	ME:MP + OC + ICAAROMA + 24P	14.00	72%
	ME:MP + OC + ICAAROMA + 24P + RIPTiDe	16.50	74%
	ME:MP + OC + ICAAROMA + 24P + 8P + RIPTiDe	25.00	58%
	ME:MP + OC + ICAAROMA + 24P + 8P	28.50	61%
	ME:MP + OC + 24P	29.50	56%
	ME:MP + OC + 24P ++ RIPTiDe	32.00	54%
Top 10 SE pipelines	SE:MP + ICAFIX + 24P	10.50	84%
	SE:MP + ICAAROMA + 24P	19.50	68%
	SE:MP + 24P + RIPTiDe	30.50	54%
	SE:MP + ICAAROMA + 24P + 8P + GMSR	35.00	51%
	SE:MP + ICAAROMA + 24P + RIPTiDe	35.50	49%
	SE:MP + ICAFIX + 24P + RIPTiDe	43.50	40%
	SE:MP + 24P	43.50	50%
	SE:MP + 24P + 8P + GMSR	45.00	38%
	SE:MP + ICAFIX + 24P + 8P	45.50	39%
	SE:MP + 24P + 8P	47.00	47%

This was computed via the average of all kernel ridge regression result averages. Censoring did not appear to make a substantial difference with regard to pipeline performance at this coarse level of measurement. MP refers to minimal preprocessing with fMRIPrep.

## DISCUSSION

In this study, we compared 90 different SE and ME pipelines with respect to six different measures of data quality in addition to evaluating their influence on the prediction of behavioral measures measured outside the scanner. We determined that ME pipelines generally outperformed SE pipelines across our denoising metrics and also yielded the highest effect sizes for behavioral prediction models. An approximate ranking scheme aggregating results across all benchmarks indicated that ME pipelines involving ICA-based denoising generally fared well. ME OC with chained ME-ICA and ICAFIX plus 24 head motion parameter, CSF, WM, and GMS regression (ME:MP + OC + MEICA + ICAFIX + 24P + 8P + GMSR) performed best when only considering denoising efficacy metrics. ME OC with only ICAFIX plus 24 head motion parameter regression (ME:MP + OC + MEICA + ICAFIX + 24P) performed the best when only considering kernel ridge regression results. However, ME OC with only ICA-AROMA plus 24 head motion parameter regression and RIPTiDe (ME:MP + OC + ICAAROMA + 24P + RIPTiDe) offered the best trade-off between denoising efficacy and behavioral prediction.

### Benchmarking Denoising Efficacy

The use of ME data was associated with performance advantages for DVARS, TSNR, and QC-FC distance dependence, consistent with past findings (Kundu et al., 2017). ME pipelines were associated with a slight increase in VE1 compared with their SE counterparts with no discernible differences in QC-FC correlations. These findings suggest that while ME acquisitions can improve SNR, they do not necessarily lead to a major improvement in the degree to which FC estimates are contaminated by motion.

For SE data, ICA-FIX outperformed ICA-AROMA and pipelines that did not use any ICA-based denoising strategy across several data quality metrics (TSNR, DVARS, QC-FC distance dependence), consistent with previous evidence that ICA-AROMA is a suboptimal denoising algorithm relative to ICA-FIX (Carone et al., 2017; Glasser et al., 2019). Among pipelines using ICA-FIX, the further application of RIPTiDe without additional WM and CSF regressors was generally associated with the best performance for VE1, TSNR, and DVARS, marginally surpassing the performance of GMSR. GMSR showed a small advantage over RIPTiDe for QC-FC. Only RIPTiDe and GMSR were successful in mitigating FCI.

In ME data, we also observed an advantage for pipelines using ICA-FIX over ME-ICA alone, ICA-AROMA and no s-ICA denoising for TSNR, DVARS, and QC-FC distance dependence. However, the best denoising performance resulted from the combination of ME-ICA and ICA-FIX. The additional use of either GMSR or RIPTiDE led to further performance gains, with the differences between the two forms of WSD removal being marginal.

Together, these findings suggest that ME pipelines improve SNR and that the combination of ME-ICA, ICA-FIX, and either RIPTiDE or GMSR results in the highest denoising efficacy. For SE data, ICA-FIX combined with either GMSR or RIPTiDE results in the best performance.

### Benchmarking Behavioral Prediction Accuracy

QCMs do not quantify the degree of behaviorally relevant variance removed during denoising. As such, a pipeline may perform well on QCM-related benchmarks simply because it is too aggressive, removing both variance relevant for specific research questions and noise. We therefore also investigated relative performance in predicting cognitive and personality measures using KRR. These analyses suggested that the more aggressive pipelines may indeed remove behaviorally relevant signal. Specifically, while the best performing pipelines according to QCMs generally incorporated GMSR or RIPTiDE, the pipelines that were most predictive of behavior did not involve WSD removal.

Previous work has demonstrated that head motion covaries with interindividual differences in behavior and that the removal of motion-associated variance from FC can reduce brain–behavior relationships (Siegel et al., 2017), while other work has shown that WSD attenuation through GSR, which covaries with head motion, can boost brain–behavior relationships (J. Li et al., 2019). These results are further complicated by evidence that GSR may only enhance brain–behavior estimates in certain datasets (Pavlovich et al., 2024). Our analyses covaried for mean FD, suggesting that the relative success of pipelines that do not incorporate WSD removal is unlikely to be explained by residual covariance between head motion and behavior. Indeed, recent evidence indicates that LFOs contain behaviorally relevant variance and that their removal may attenuate FC-based predictions of behavior (Welsh et al., 2025). Together, these mixed findings suggest that behavioral prediction accuracy on its own may be an insufficiently sensitive measure of the ability of a pipeline to preserve useful signal and more sophisticated methods may be required (e.g., Aquino et al., 2020; Glasser et al., 2019).

Some recent work indicates that FC estimates derived from task-based fMRI-derived FC more accurately predict behavior than resting-state FC estimates (Zhao et al., 2023), although this result has been challenged elsewhere (Korponay et al., 2025). Further research is therefore needed to optimize in-scanner tasks to derive maximally informative information given a specific research question. As such, resting-state designs may not provide an optimal means for comparing predictive models of individual differences in behavior. Although relatively underacknowledged, measurement error in estimating interindividual differences in behavior also imposes a significant limit on achieving robust and reliable brain–behavior associations. The adoption of statistical and psychometric approaches for deriving more precise estimates of behavior may help improve effect sizes (Tiego et al., 2023). Indeed, the prediction accuracies we observed were generally small (i.e., all *r* < .15) and differences between the top 4 pipelines and the next best-performing pipelines were on the order of *r* < .05, so the gains associated with choosing one particular pipeline over another are modest.

### A Trade-Off Between Noise Removal and Signal Preservation?

No single pipeline simultaneously maximized data quality and behavioral prediction accuracy, which aligns with past work in SE datasets (Pavlovich et al., 2024). To discern overall pipeline performance, we generated overall rankings for the QCMs and behavioral prediction independently, before subsequently computing an overall ranking from these composites (see Table 1). We determined that SE pipelines generally underperformed relative to ME pipelines on these measures. We propose that ME sequences may present a more effective approach than SE sequences for studying brain–behavior associations with rs-fMRI while balancing denoising concerns. Optimal combination of ME data followed by ICA-AROMA, 24P regression, and RIPTiDe generally provided strong denoising efficacy while maintaining robust brain–behavior associations for ME data. However, we emphasize that our ranking scheme should be used as a heuristic only and that investigators should consider whether they prioritize optimizing certain benchmarks over others. For example, ME combination with ME-ICA and ICA-FIX (with additional head motion parameter, CSF, WM, and GMS regression applied) was considered the best performing pipeline regarding denoising efficacy alone, and ME combination with ME-ICA and ICA-FIX (with head motion parameter regression) was considered optimal purely based on the FC-based behavioral kernel ridge regression model results.

### Limitations

Further work is needed to validate our tentative recommendations using independent datasets, particularly in light of research highlighting variability in pipeline performance across different datasets (Pavlovich et al., 2024). Future studies should investigate the factors contributing to variability in prediction and denoising performance across pipelines in different datasets.

Our study used a common minimal preprocessing pipeline. Recent evidence indicates that variations in these earlier steps can also impact FC estimates (Li et al., 2024). Understanding how choices at early processing stages interact with denoising strategies will be an important extension of this work. We also used common mixing matrices derived from FSL for all ICA approaches, which allowed us to easily combine different ICA-based approaches (e.g., ME-ICA and ICA-FIX). This implementation is distinct from the default approach in *tedana* and should therefore be considered when interpreting our findings. A further consideration is that ICA-based approaches incorporate a stochastic element that can yield variable results from run to run (Pavlovich et al., 2024). We did not investigate this variability here to limit computational burden, but it should be considered in any future applications.

Our SE pipelines used SE data extracted from an ME sequence (e.g., also see Dipasquale et al., 2017, for an example of this practice). It has recently been argued that an optimized SE acquisition could achieve shorter TRs by, for example, circumventing the need to sample echoes 3 and 4, making a direct comparison between acquisition types “fairer” and more generalizable (Giubergia et al., 2025). In our case, however, the TR was already < 1 s, which is comparable with current state-of-the-art rs-fMRI imaging protocols. Further reductions in TR possible with purely SE acquisitions are thus likely to be minor and may not have a major impact on data quality (Bhandari et al., 2020; Chen et al., 2019; Demetriou et al., 2018; Dowdle et al., 2021). Moreover, differences in TR and temporal degrees of freedom between SE and ME acquisitions make it difficult to determine whether any pipeline differences are attributable to variations in the number of echoes acquired at each TR or the number of volumes in the acquisition. A comparison of our approach with an SE-optimized acquisition could help disentangle these possibilities.

Future studies could also evaluate within-subject network reproducibility in highly sampled individuals as an additional means of comparing pipelines. The total scan duration in the present study exceeds the common 5- to 10-min acquisition protocols in most fMRI studies and approximates the point at which further gains in within-subject reproducibility for SE acquisitions begin to plateau (Birn et al., 2013; Nakuci et al., 2023). Moreover, ME acquisitions can achieve comparable reliability to SE acquisition schemes with only about one third of the scan time (Lynch et al., 2020). Critically, FC estimates exhibiting high reliability may not necessarily encode behaviorally meaningful information (Noble et al., 2017). Within-subject reproducibility could serve as a useful complementary metric in future work, but but there may be some caveats to its use.

Finally, an alternative approach to estimating preservation of neural signal could involve examining the alignment between FC and structural connectivity (Du & Buckner, 2021; Miranda-Dominguez et al., 2014). However, in humans, diffusion tractography, which is currently the only available technique for noninvasive quantification of structural connectivity, has known limitations (Maier-Hein et al., 2017; Schilling et al., 2019; Thomas et al., 2014) and is also susceptible to variations in preprocessing and analysis strategies (Gajwani et al., 2023; Oldham et al., 2020; Schilling et al., 2021). Moreover, correlation-based FC estimates capture direct and indirect (polysynaptic) interactions between regions, whereas structural connectivity only quantifies the former (Goñi et al., 2014; Honey et al., 2009; Zalesky et al., 2012).

### Conclusions

We compared denoising approaches for ME and SE fMRI acquisitions, along with various techniques to manage WSDs (e.g., GMSR, RIPTiDe) following s-ICA. We tested 90 different pipelines across six established data quality metrics and evaluated their capacity to predict personality and cognition using resting-state functional connectivity (FC). Our results indicated that no single pipeline maximizes efficacy and behavioral prediction accuracies. However, balancing both QCM results and relative behavioral prediction performance, the application of ICA-AROMA, 24P regression, and RIPTiDe to ME data resulted in the best aggregate outcomes. In general, ME acquisitions appear superior to SE data.

## Supporting Information

Supporting information for this article is available at https://doi.org/10.1162/NETN.a.547.

## Author Contributions

Toby Constable: Conceptualization; Formal analysis; Investigation; Methodology; Validation; Visualization; Writing – original draft; Writing – review & editing. Jeggan Tiego: Conceptualization; Investigation; Methodology; Supervision; Writing – original draft; Writing – review & editing. Kane Pavlovich: Formal analysis; Investigation; Methodology; Validation; Writing – review & editing. Arshiya Sangchooli: Formal analysis; Validation; Writing – review & editing. Priscilla Thalenberg Levi: Formal analysis; Validation. Bree Hartshorn: Data curation; Investigation; Project administration. Jessica Kwee: Data curation; Investigation; Project administration. Kate Fortune: Data curation; Project administration. Kate Thompson: Data curation; Project administration. Sam Brown: Data curation; Project administration. James McLauchlan: Data curation; Project administration. Nancy Ong Tran: Data curation; Project administration. Rebecca O’Neill: Data curation; Project administration. Mark A. Bellgrove: Investigation; Project administration; Resources; Writing – review & editing. Alex Fornito: Conceptualization; Data curation; Investigation; Methodology; Project administration; Supervision; Writing – original draft; Writing – review & editing.

## Ethics

The study was approved by the Monash Human Research Ethics Committee (Project ID: 12692 & 43629).

## Code Availability

Project code can be found here: https://github.com/cicadawing/Single-vs-Multi-Echo-fMRI-Denoising-Strategies.

## Supplementary Material



## TECHNICAL TERMS

24P regression: A nuisance regression approach designed to mitigate motion-related confounds by regressing out the six rigid-body motion parameters estimated during realignment, along with their temporal derivatives and quadratic expansions.
8P regression: A nuisance regression approach designed to mitigate white matter and cerebrospinal-fluid-based confounds by regressing out average time-courses within each tissue compartment, along with associated temporal derivatives and quadratic expansions.
Gray matter signal regression (GMSR): A nuisance regression approach designed to mitigate global noise fluctuations by regressing out the average signal within the gray matter tissue compartment, optionally also including associated temporal derivatives and quadratic expansions.
Regressor Interpolation at Progressive Time Delays (RIPTiDE) algorithm: An algorithm that mitigates blood arrival time effects in fMRI data to better isolate neuronal signal from physiological noise.
Spatial independent component analysis (s-ICA): A dimensionality reduction approach that decomposes spatiotemporal signals into spatially independent sources. Each source may represent noise, neuronal signal, or a mixture of both.
T_2_* rate: The rate at which signals decay in intensity post RF pulse. This information can be estimated and leveraged in ME but not in SE acquisitions.
Spike regression (SR): The process of identifying and censoring (controlling for) time points during fMRI acquisition that are characterized by excessive head motion.
Functional connectivity: The statistical dependence of spatially distinct neurophysiological signals, typically estimated via the product–moment correlation between regional mean time series.
Multi-echo (ME) data: ME fMRI protocols acquire multiple images following a single radiofrequency (RF) pulse, in contrast to standard single-echo (SE) acquisitions. In ME protocols, each read-out is associated with varying contrast levels: Earlier echoes have higher signal intensity and are associated with reduced signal dropout due to their proximity to the RF pulse, whereas later echoes generally provide increased tissue contrast but lower signal intensity.
