# Supplementary material for: An evaluation of the efficacy of single-echo and multi-echo fMRI denoising strategies
Source: Netw Neurosci. 2026 Apr 22;10(2):444–74. doi: 10.1162/NETN.a.547 (PMC13108510; doi:10.1162/NETN.a.547)
Supplement: Supplementary file 1 [file netn-10-2-444-s001.pdf]

ECHO	Pipeline	Censoring Status	Ranked VE1	Percentage Decr VE1	Ranked DVARS	Percentage Decr DVARS	Ranked TSNR	Percentage Decr TSNR	Rank QCFC (Uncorr)	Percentage Decr QCFC (Uncorr)	Rank QCFC (Corr)	Percentage Decr QCFC (Corr)	Rank FCI (Binarised)	Percentage Decr FCI (Binarised)	Rank QC-FC Distance Correlation	Percentage Decr QC-FC Distance Correlation	Overall Denoising Efficacy Rank	Overall Denoising Efficacy Rank - Not Considering FCI	Overall Denoising Efficacy Percent Decr	Overall Denoising Efficacy Percent Decr - Not Considering FCI	Rank BFI Value Prediction	Percentage Decr Mean BFI Value Prediction	Rank WASI Value Prediction	Percentage Decr Mean WASI Value Prediction	Overall Prediction Rank	Overall Prediction Percentage Decr	Overall Rank	Overall Percentage Decr	Overall Rank - Not Considering FCI	Overall Percentage Decr - Not Considering FCI
ME	ME:MP+OC+ICAAROMA+24P+RIPTiDe	Censoring Applied	27	86.11%	43	73.56%	51	45.07%	14	98.00%	13	99.72%	1	100%	73	40.33%	31.71	36.10	77.54%	74.33%	12	74.83%	19	76.24%	15.5	75.53%	23.61	76.54%	25.80	74.93%
ME	ME:MP+OC+ICAAROMA+24P+RIPTiDe	No Censoring Applied	28	86.04%	44	73.39%	52	44.96%	15	97.96%	12	99.72%	1	100%	74	39.98%	32.29	36.76	77.44%	74.21%	11	75.07%	22	73.66%	16.5	74.36%	24.39	75.90%	26.63	74.29%
ME	ME:MP+OC+MEICA+ICAAROMA+24P+RIPTiDe	Censoring Applied	44	79.34%	21	92.70%	13	88.31%	39	87.56%	42	93.81%	1	100%	2	99.53%	23.14	26.31	91.61%	90.41%	54	29.87%	40	55.72%	47	42.79%	35.07	67.20%	36.65	66.60%
ME	ME:MP+OC+ICAFIX+24P	No Censoring Applied	88	0.41%	36	79.59%	40	52.63%	80	7.91%	80	7.92%	2	0%	12	93.62%	48.29	54.90	34.58%	39.52%	1	100.00%	14	84.81%	7.5	92.40%	27.89	63.49%	31.20	65.96%
ME	ME:MP+OC+ICAFIX+24P	Censoring Applied	87	0.48%	35	79.74%	39	52.78%	79	8.02%	79	8.04%	2	0%	16	92.94%	48.14	54.73	34.57%	39.51%	2	98.81%	13	85.43%	7.5	92.12%	27.82	63.34%	31.12	65.81%
ME	ME:MP+OC+ICAAROMA+24P+8P+RIPTiDe	Censoring Applied	31	84.20%	41	75.96%	47	47.92%	61	80.22%	60	90.21%	1	100%	64	53.93%	43.57	49.65	76.06%	72.64%	31	40.63%	17	76.56%	24	58.59%	33.79	67.33%	36.83	65.62%
ME	ME:MP+OC+ICAAROMA+24P+8P+RIPTiDe	No Censoring Applied	32	84.18%	42	75.78%	48	47.80%	64	79.92%	64	89.70%	1	100%	63	54.68%	44.86	51.12	76.01%	72.58%	29	42.03%	21	74.82%	25	58.42%	34.93	67.22%	38.06	65.50%
ME	ME:MP+OC+MEICA+ICAAROMA+24P+8P+GMSR	Censoring Applied	33	84.04%	19	93.23%	23	76.20%	6	99.44%	1	100.00%	1	100%	57	60.14%	20.00	22.71	87.58%	85.81%	46	33.53%	43	52.42%	44.5	42.97%	32.25	65.28%	33.61	64.39%
ME	ME:MP+OC+MEICA+ICAAROMA+24P+RIPTiDe	No Censoring Applied	43	79.35%	22	92.55%	14	88.13%	40	87.46%	45	93.59%	1	100%	1	100.00%	23.71	26.96	91.58%	90.38%	71	20.73%	39	55.93%	55	38.33%	39.36	64.96%	40.98	64.36%
ME	ME:MP+OC+MEICA+ICAFIX+24P	No Censoring Applied	86	1.75%	34	84.00%	34	59.89%	84	2.32%	84	2.38%	2	0%	44	74.08%	52.57	59.80	32.06%	36.64%	3	87.77%	6	92.91%	4.5	90.34%	28.54	61.20%	32.15	63.49%
ME	ME:MP+OC+MEICA+ICAFIX+24P	Censoring Applied	85	1.81%	33	84.14%	33	60.04%	83	2.43%	83	2.51%	2	0%	47	73.33%	52.29	59.47	32.04%	36.61%	4	86.49%	5	93.84%	4.5	90.16%	28.39	61.10%	31.98	63.39%
ME	ME:MP+OC+MEICA+ICAAROMA+24P+8P+GMSR	No Censoring Applied	34	84.00%	20	93.10%	24	76.03%	8	99.40%	2	99.99%	1	100%	58	59.98%	21.00	23.86	87.50%	85.71%	52	30.94%	44	50.93%	48	40.94%	34.50	64.22%	35.93	63.32%
ME	ME:MP+OC+24P+RIPTiDe	Censoring Applied	21	89.17%	49	67.74%	57	37.15%	24	95.88%	28	98.65%	1	100%	85	16.70%	37.86	43.12	72.18%	68.21%	21	54.42%	36	61.25%	28.5	57.84%	33.18	65.01%	35.81	63.02%
SE	SE:MP+ICAFIX+24P	Censoring Applied	73	22.62%	75	37.95%	55	37.98%	73	29.37%	73	32.22%	2	0%	13	93.49%	52.00	59.14	36.23%	41.41%	6	84.96%	15	81.63%	10.5	83.29%	31.25	59.76%	34.82	62.35%
SE	SE:MP+ICAFIX+24P	No Censoring Applied	74	22.54%	76	37.79%	56	37.87%	74	28.37%	74	30.90%	2	0%	22	90.03%	54.00	61.43	35.36%	40.41%	5	85.78%	16	81.58%	10.5	83.68%	32.25	59.52%	35.96	62.04%
SE	SE:MP+24P+RIPTiDe	Censoring Applied	3	99.61%	77	35.96%	69	28.39%	21	96.73%	23	99.16%	1	100%	79	30.55%	39.00	44.43	70.06%	65.78%	17	61.63%	41	54.58%	29	58.11%	34.00	64.08%	36.71	61.94%
ME	ME:MP+OC+MEICA+ICAFIX+24P+8P+GMSR	Censoring Applied	37	82.00%	3	99.30%	11	88.60%	1	100.00%	3	99.97%	1	100%	8	96.47%	9.14	10.31	95.19%	94.51%	60	26.01%	67	32.33%	63.5	29.17%	36.32	62.18%	36.90	61.84%
ME	ME:MP+OC+MEICA+ICAAROMA+24P+8P	Censoring Applied	70	61.99%	29	87.90%	29	67.69%	50	84.04%	43	93.76%	2	0%	37	79.58%	37.14	42.16	67.85%	77.54%	70	20.94%	29	70.63%	49.5	45.79%	43.32	56.82%	45.83	61.66%
SE	SE:MP+ICAFIX+24P+RIPTiDe	Censoring Applied	15	91.30%	57	60.35%	37	54.27%	4	99.82%	15	99.48%	1	100%	28	84.83%	22.43	25.49	84.29%	82.05%	23	48.72%	64	33.40%	43.5	41.06%	32.96	62.68%	34.49	61.56%
ME	ME:MP+OC+MEICA+ICAAROMA+24P	Censoring Applied	79	3.18%	37	77.60%	41	52.42%	87	0.06%	88	0.06%	2	0%	32	81.79%	52.29	59.47	30.73%	35.12%	9	75.89%	1	100.00%	5	87.94%	28.64	59.34%	32.23	61.53%
ME	ME:MP+OC+MEICA+ICAAROMA+24P	No Censoring Applied	81	3.11%	38	77.43%	42	52.27%	88	0.05%	87	0.08%	2	0%	30	83.01%	52.57	59.80	30.85%	35.26%	10	75.84%	2	99.17%	6	87.50%	29.29	59.18%	32.90	61.38%
SE	SE:MP+ICAFIX+24P+RIPTiDe	No Censoring Applied	16	91.26%	58	60.23%	38	54.17%	2	99.97%	16	99.45%	1	100%	27	86.78%	22.57	25.65	84.55%	82.34%	34	38.81%	53	41.96%	43.5	40.38%	33.04	62.47%	34.58	61.36%
ME	ME:MP+OC+MEICA+24P+RIPTiDe	Censoring Applied	39	80.74%	25	92.21%	15	87.92%	33	89.76%	37	95.07%	1	100%	5	98.53%	22.14	25.16	92.03%	90.89%	61	25.48%	56	37.99%	58.5	31.73%	40.32	61.88%	41.83	61.31%
ME	ME:MP+OC+MEICA+24P	Censoring Applied	80	3.15%	39	76.46%	45	49.62%	89	0.00%	90	0.00%	2	0%	26	86.99%	53.00	60.29	30.89%	35.30%	7	82.93%	7	91.70%	7	87.31%	30.00	59.10%	33.64	61.31%
ME	ME:MP+OC+ICAAROMA+24P+8P+GMSR	Censoring Applied	23	88.15%	45	73.19%	61	35.36%	17	97.56%	17	99.44%	1	100%	84	20.76%	35.43	40.35	73.49%	69.71%	40	36.81%	32	68.84%	36	52.82%	35.71	63.16%	38.17	61.27%
ME	ME:MP+OC+MEICA+ICAAROMA+24P+8P	No Censoring Applied	69	62.00%	30	87.75%	30	67.53%	49	84.28%	41	93.97%	2	0%	38	78.90%	37.00	42.00	67.78%	77.46%	69	21.55%	33	67.97%	51	44.76%	44.00	56.27%	46.50	61.11%
ME	ME:MP+OC+MEICA+24P	No Censoring Applied	82	3.09%	40	76.29%	46	49.48%	90	0.00%	89	0.06%	2	0%	24	87.94%	53.29	60.61	30.98%	35.41%	8	82.24%	8	91.15%	8	86.69%	30.64	58.84%	34.31	61.05%
ME	ME:MP+OC+24P+8P+RIPTiDe	Censoring Applied	26	86.45%	47	70.10%	53	39.40%	67	78.12%	67	87.15%	1	100%	76	35.30%	48.14	54.88	70.93%	66.78%	38	37.26%	25	72.51%	31.5	54.88%	39.82	62.91%	43.19	60.83%
ME	ME:MP+OC+MEICA+ICAFIX+24P+8P+GMSR	No Censoring Applied	38	81.99%	4	99.20%	12	88.43%	3	99.89%	4	99.94%	1	100%	7	96.94%	9.86	11.12	95.20%	94.51%	62	24.98%	69	29.25%	65.5	27.12%	37.68	61.16%	38.31	60.81%
SE	SE:MP+ICAAROMA+24P+RIPTiDe	No Censoring Applied	10	97.32%	72	42.31%	64	33.49%	13	98.48%	5	99.91%	1	100%	60	59.12%	32.14	36.59	75.80%	72.35%	22	53.41%	49	44.02%	35.5	48.72%	33.82	62.26%	36.05	60.53%
ME	ME:MP+OC+ICAAROMA+24P+8P	No Censoring Applied	62	62.99%	52	66.29%	68	30.10%	57	81.82%	54	92.26%	2	0%	78	34.87%	53.29	60.61	52.62%	60.13%	45	34.78%	12	86.92%	28.5	60.85%	40.89	56.74%	44.56	60.49%
ME	ME:MP+OC+24P+8P+RIPTiDe	No Censoring Applied	25	86.53%	48	69.92%	54	39.30%	68	77.63%	68	86.68%	1	100%	75	36.15%	48.43	55.20	70.89%	66.73%	36	37.52%	28	70.65%	32	54.08%	40.21	62.49%	43.60	60.41%
ME	ME:MP+OC+ICAAROMA+24P+8P+GMSR	No Censoring Applied	24	88.12%	46	73.01%	62	35.26%	18	97.53%	18	99.43%	1	100%	83	21.01%	36.00	41.00	73.48%	69.69%	43	35.81%	34	65.59%	38.5	50.70%	37.25	62.09%	39.75	60.19%
SE	SE:MP+24P+RIPTiDe	No Censoring Applied	4	99.58%	78	35.77%	70	28.31%	22	96.63%	25	99.04%	1	100%	80	29.50%	40.00	45.57	69.83%	65.52%	24	48.64%	37	60.31%	30.5	54.48%	35.25	62.15%	38.04	60.00%
ME	ME:MP+OC+ICAAROMA+24P+8P	Censoring Applied	61	63.03%	51	66.48%	66	30.20%	55	81.88%	53	92.39%	2	0%	77	35.27%	52.14	59.31	52.75%	60.29%	51	31.51%	11	87.13%	31	59.32%	41.57	56.04%	45.15	59.80%

ECHO	Pipeline	Censoring Status	Ranked VE1	Percentage Decr VE1	Ranked DVARS	Percentage Decr DVARS	Ranked TSNR	Percentage Decr TSNR	Rank QCFC (Uncorr)	Percentage Decr QCFC (Uncorr)	Rank QCFC (Corr)	Percentage Decr QCFC (Corr)	Rank FCI (Binarised)	Percentage Decr FCI (Binarised)	Rank QC-FC Distance Correlation	Percentage Decr QC-FC Distance Correlation	Overall Denoising Efficacy Rank	Overall Denoising Efficacy Rank - Not Considering FCI	Overall Denoising Efficacy Percent Decr	Overall Denoising Efficacy Percent Decr - Not Considering FCI	Rank BFI Value Prediction	Percentage Decr Mean BFI Value Prediction	Rank WASI Value Prediction	Percentage Decr Mean WASI Value Prediction	Overall Prediction Rank	Overall Prediction Percentage Decr	Overall Rank	Overall Percentage Decr	Overall Rank - Not Considering FCI	Overall Percentage Decr - Not Considering FCI
ME	ME:MP+OC+24P+RIPTiDe	No Censoring Applied	22	89.03%	50	67.54%	58	37.05%	23	95.98%	27	98.73%	1	100%	88	15.09%	38.43	43.78	71.92%	67.91%	25	48.52%	42	53.90%	33.5	51.21%	35.96	61.56%	38.64	59.56%
SE	SE:MP+ICAAROMA+24P+8P+GMSR	Censoring Applied	5	99.35%	79	30.50%	73	23.85%	19	97.22%	20	99.28%	1	100%	70	42.13%	38.14	43.45	70.33%	66.09%	14	63.59%	52	42.22%	33	52.91%	35.57	61.62%	38.22	59.50%
ME	ME:MP+OC+MEICA+24P+8P+GMSR	Censoring Applied	29	85.14%	23	92.40%	25	73.42%	11	98.72%	10	99.73%	1	100%	68	48.64%	23.86	27.12	85.43%	83.35%	42	36.16%	61	34.55%	51.5	35.35%	37.68	60.39%	39.31	59.35%
ME	ME:MP+OC+MEICA+ICAAROMA+24P+8P+RIPTiDe	Censoring Applied	46	79.07%	13	94.50%	5	92.18%	60	80.50%	61	90.09%	1	100%	11	93.89%	28.14	32.02	90.03%	88.61%	82	14.35%	45	45.43%	63.5	29.89%	45.82	59.96%	47.76	59.25%
ME	ME:MP+OC+MEICA+24P+RIPTiDe	No Censoring Applied	40	80.71%	26	92.07%	17	87.74%	34	89.64%	38	94.99%	1	100%	6	98.00%	23.14	26.31	91.88%	90.72%	72	19.02%	58	36.08%	65	27.55%	44.07	59.71%	45.65	59.13%
SE	SE:MP+ICAAROMA+24P+RIPTiDe	Censoring Applied	9	97.33%	71	42.50%	63	33.57%	16	97.77%	14	99.71%	1	100%	55	60.54%	32.71	37.24	75.92%	72.48%	19	55.27%	60	35.44%	39.5	45.35%	36.11	60.64%	38.37	58.92%
ME	ME:MP+OC+MEICA+ICAAROMA+24P+8P+RIPTiDe	No Censoring Applied	45	79.08%	14	94.36%	6	92.00%	58	81.66%	57	91.10%	1	100%	15	92.95%	28.00	31.86	90.16%	88.76%	84	13.29%	46	44.60%	65	28.94%	46.50	59.55%	48.43	58.85%
SE	SE:MP+ICAAROMA+24P+8P+GMSR	No Censoring Applied	6	99.25%	80	30.29%	74	23.76%	20	97.13%	22	99.23%	1	100%	69	42.51%	38.86	44.27	70.31%	66.07%	16	63.21%	54	39.79%	35	51.50%	36.93	60.90%	39.63	58.78%
ME	ME:MP+OC+ICAFIX+24P+8P+GMSR	Censoring Applied	36	83.09%	9	95.96%	19	80.29%	9	99.15%	8	99.74%	1	100%	14	93.29%	13.71	15.53	93.07%	92.09%	49	31.95%	81	16.89%	65	24.42%	39.36	58.75%	40.27	58.25%
ME	ME:MP+OC+MEICA+24P+8P+GMSR	No Censoring Applied	30	85.12%	24	92.26%	26	73.26%	12	98.67%	7	99.74%	1	100%	67	48.67%	23.86	27.12	85.39%	83.30%	47	33.30%	66	32.92%	56.5	33.11%	40.18	59.25%	41.81	58.21%
ME	ME:MP+OC+24P+8P+GMSR	Censoring Applied	19	90.80%	53	65.97%	71	24.97%	29	94.88%	31	98.32%	1	100%	90	0.00%	42.00	47.86	67.85%	63.26%	27	43.94%	35	61.66%	31	52.80%	36.50	60.32%	39.43	58.03%
SE	SE:MP+ICAFIX+24P+8P+GMSR	Censoring Applied	13	95.50%	63	51.33%	43	52.11%	5	99.54%	6	99.81%	1	100%	4	99.09%	19.29	21.90	85.34%	83.25%	35	38.70%	71	25.68%	53	32.19%	36.14	58.77%	37.45	57.72%
ME	ME:MP+OC+ICAFIX+24P+8P+GMSR	No Censoring Applied	35	83.09%	10	95.84%	20	80.12%	10	98.93%	11	99.72%	1	100%	17	92.86%	14.86	16.84	92.94%	91.93%	50	31.89%	86	13.42%	68	22.66%	41.43	57.80%	42.42	57.29%
ME	ME:MP+OC+24P+8P+GMSR	No Censoring Applied	20	90.79%	54	65.78%	72	24.88%	30	94.81%	32	98.30%	1	100%	89	1.22%	42.57	48.51	67.97%	63.39%	28	42.90%	38	58.40%	33	50.65%	37.79	59.31%	40.76	57.02%
SE	SE:MP+ICAFIX+24P+8P+GMSR	No Censoring Applied	14	95.47%	64	51.20%	44	52.01%	7	99.41%	9	99.74%	1	100%	3	99.25%	20.29	23.04	85.30%	83.20%	37	37.34%	72	24.31%	54.5	30.82%	37.39	58.06%	38.77	57.01%
SE	SE:MP+ICAFIX+24P+8P	No Censoring Applied	58	66.88%	68	45.24%	50	46.06%	42	87.11%	36	97.18%	2	0%	10	94.32%	38.00	43.14	62.40%	71.31%	26	45.01%	65	33.17%	45.5	39.09%	41.75	50.74%	44.32	55.20%
ME	ME:MP+OC+ICAFIX+24P+RIPTiDe	Censoring Applied	47	78.92%	11	94.91%	16	87.80%	38	87.66%	44	93.61%	1	100%	36	79.94%	27.57	31.37	88.98%	87.40%	58	28.56%	82	16.66%	70	22.61%	48.79	55.79%	50.68	55.00%
ME	ME:MP+OC+MEICA+ICAFIX+24P+RIPTiDe	Censoring Applied	54	77.41%	5	98.01%	3	95.29%	52	83.08%	63	90.05%	1	100%	54	62.69%	33.14	37.73	86.65%	84.74%	73	18.84%	68	31.36%	70.5	25.10%	51.82	55.88%	54.12	54.92%
ME	ME:MP+OC+ICAFIX+24P+RIPTiDe	No Censoring Applied	49	78.90%	12	94.78%	18	87.61%	37	87.68%	46	93.50%	1	100%	35	79.98%	28.29	32.18	88.92%	87.34%	59	26.09%	76	18.51%	67.5	22.30%	47.89	55.61%	49.84	54.82%
SE	SE:MP+ICAFIX+24P+8P	Censoring Applied	57	66.92%	67	45.38%	49	46.17%	41	87.35%	35	97.34%	2	0%	9	94.99%	37.14	42.16	62.59%	71.54%	30	41.93%	62	33.76%	46	37.85%	41.57	50.22%	44.08	54.69%
ME	ME:MP+OC+MEICA+24P+8P	Censoring Applied	66	62.78%	31	87.05%	31	64.97%	54	82.78%	51	93.01%	2	0%	48	73.29%	40.43	45.92	66.27%	75.73%	66	22.38%	48	44.44%	57	33.41%	48.71	49.84%	51.46	54.57%
ME	ME:MP+OC+MEICA+24P+8P	No Censoring Applied	65	62.80%	32	86.90%	32	64.82%	53	83.07%	48	93.30%	2	0%	50	72.64%	40.29	45.76	66.22%	75.68%	64	23.05%	51	43.78%	57.5	33.41%	48.89	49.81%	51.63	54.54%
ME	ME:MP+OC+MEICA+ICAFIX+24P+RIPTiDe	No Censoring Applied	53	77.44%	6	97.90%	4	95.11%	51	83.17%	62	90.09%	1	100%	53	63.34%	32.86	37.41	86.72%	84.83%	76	17.62%	70	28.06%	73	22.84%	52.93	54.78%	55.20	53.83%
SE	SE:MP+ICAAROMA+24P+8P+RIPTiDe	Censoring Applied	12	96.27%	69	44.89%	59	35.77%	47	84.52%	50	93.08%	1	100%	42	74.73%	40.00	45.57	75.61%	72.12%	44	35.12%	59	35.77%	51.5	35.44%	45.75	55.53%	48.54	53.78%
ME	ME:MP+OC+ICAFIX+24P+8P+RIPTiDe	Censoring Applied	50	78.81%	7	96.85%	8	91.84%	62	80.03%	59	90.41%	1	100%	31	82.68%	31.14	35.45	88.66%	87.04%	67	22.24%	83	16.40%	75	19.32%	53.07	53.99%	55.22	53.18%
ME	ME:MP+OC+ICAFIX+24P+8P+RIPTiDe	No Censoring Applied	48	78.91%	8	96.73%	10	91.66%	62	80.03%	58	90.42%	1	100%	29	83.18%	30.86	35.12	88.71%	87.09%	65	22.96%	85	13.95%	75	18.45%	52.93	53.58%	55.06	52.77%
SE	SE:MP+ICAAROMA+24P+8P+RIPTiDe	No Censoring Applied	11	96.28%	70	44.71%	60	35.68%	48	84.44%	52	92.95%	1	100%	41	74.88%	40.43	46.06	75.56%	72.07%	48	32.01%	63	33.53%	55.5	32.77%	47.96	54.17%	50.78	52.42%
ME	ME:MP+OC+MEICA+24P+8P+RIPTiDe	Censoring Applied	42	79.79%	17	94.13%	7	91.93%	66	78.73%	66	88.46%	1	100%	20	91.39%	31.29	35.61	89.20%	87.66%	81	14.38%	75	18.97%	78	16.67%	54.64	52.94%	56.81	52.17%
ME	ME:MP+OC+MEICA+24P+8P+RIPTiDe	No Censoring Applied	41	79.85%	18	93.99%	9	91.73%	65	79.61%	65	89.40%	1	100%	21	90.66%	31.43	35.78	89.32%	87.80%	83	13.56%	77	18.35%	80	15.95%	55.71	52.64%	57.89	51.87%
ME	ME:MP+OC+MEICA+ICAFIX+24P+8P	Censoring Applied	71	61.66%	15	94.24%	21	78.56%	31	92.45%	19	99.30%	2	0%	25	87.79%	26.29	29.76	73.43%	83.92%	78	16.83%	74	19.69%	76	18.26%	51.14	45.85%	52.88	51.09%
ME	ME:MP+OC+MEICA+ICAFIX+24P+8P	No Censoring Applied	72	61.66%	16	94.13%	22	78.40%	32	92.35%	21	99.24%	2	0%	23	88.30%	26.86	30.41	73.44%	83.93%	74	18.31%	78	18.15%	76	18.23%	51.43	45.83%	53.20	51.08%
ME	ME:MP+OC+ICAFIX+24P+8P	No Censoring Applied	68	62.33%	28	90.37%	28	70.27%	36	89.14%	34	98.06%	2	0%	19	91.60%	30.71	34.82	71.68%	81.92%	63	23.99%	84	15.76%	73.5	19.87%	52.11	45.78%	54.16	50.90%
ME	ME:MP+OC+ICAFIX+24P+8P	Censoring Applied	67	62.34%	27	90.49%	27	70.43%	35	89.22%	33	98.15%	2	0%	18	92.13%	29.86	33.84	71.82%	82.08%	68	21.56%	80	17.38%	74	19.47%	51.93	45.65%	53.92	50.78%
ME	ME:MP+OC+24P+8P	No Censoring Applied	64	62.95%	60	58.51%	78	20.51%	59	81.57%	56	91.58%	2	0%	87	16.09%	58.00	66.00	47.32%	54.08%	75	18.00%	24	72.83%	49.5	45.41%	53.75	46.37%	57.75	49.75%
ME	ME:MP+OC+24P+8P	Censoring Applied	63	62.98%	59	58.72%	77	20.60%	56	81.85%	55	91.79%	2	0%	86	16.11%	56.86	64.69	47.44%	54.21%	77	17.44%	23	72.83%	50	45.13%	53.43	46.29%	57.35	49.67%

ECHO	Pipeline	Censoring Status	Ranked VE1	Percentage Decr VE1	Ranked DVARS	Percentage Decr DVARS	Ranked TSNR	Percentage Decr TSNR	Rank QCFC (Uncorr)	Percentage Decr QCFC (Uncorr)	Rank QCFC (Corr)	Percentage Decr QCFC (Corr)	Rank FCI (Binarised)	Percentage Decr FCI (Binarised)	Rank QC-FC Distance Correlation	Percentage Decr QC-FC Distance Correlation	Overall Denoising Efficacy Rank	Overall Denoising Efficacy Rank - Not Considering FCI	Overall Denoising Efficacy Percent Decr	Overall Denoising Efficacy Percent Decr - Not Considering FCI	Rank BFI Value Prediction	Percentage Decr Mean BFI Value Prediction	Rank WASI Value Prediction	Percentage Decr Mean WASI Value Prediction	Overall Prediction Rank	Overall Prediction Percentage Decr	Overall Rank	Overall Percentage Decr	Overall Rank - Not Considering FCI	Overall Percentage Decr - Not Considering FCI
ME	ME:MP+OC+MEICA+ICAFIX+24P+8P+RIPTiDe	No Censoring Applied	51	78.14%	2	99.89%	2	99.81%	45	84.98%	39	94.55%	1	100%	51	66.87%	27.29	31.04	89.18%	87.63%	87	8.96%	87	12.31%	87	10.64%	57.14	49.91%	59.02	49.13%
ME	ME:MP+OC+MEICA+ICAFIX+24P+8P+RIPTiDe	Censoring Applied	52	78.07%	1	100.00%	1	100.00%	46	84.93%	40	94.50%	1	100%	52	66.72%	27.57	31.37	89.18%	87.63%	88	8.38%	88	12.26%	88	10.32%	57.79	49.75%	59.68	48.98%
ME	ME:MP+OC+ICAAROMA+24P	No Censoring Applied	84	2.90%	62	54.55%	76	22.02%	86	1.86%	86	1.94%	2	0%	45	73.65%	63.00	71.71	22.42%	25.62%	18	55.90%	10	88.63%	14	72.27%	38.50	47.34%	42.86	48.94%
ME	ME:MP+OC+ICAAROMA+24P	Censoring Applied	83	2.97%	61	54.76%	75	22.12%	85	1.87%	85	1.95%	2	0%	43	74.57%	62.00	70.57	22.61%	25.83%	20	54.86%	9	88.98%	14.5	71.92%	38.25	47.26%	42.54	48.88%
SE	SE:MP+24P+8P+GMSR	Censoring Applied	1	100.00%	83	17.96%	85	6.70%	27	94.93%	29	98.38%	1	100%	81	22.45%	43.86	49.98	62.92%	57.62%	32	40.02%	55	38.88%	43.5	39.45%	43.68	51.18%	46.74	48.53%
SE	SE:MP+ICAFIX+24P+8P+RIPTiDe	Censoring Applied	17	91.18%	55	62.08%	35	56.36%	25	95.14%	24	99.09%	1	100%	33	81.10%	27.14	30.88	83.56%	81.22%	86	9.28%	73	20.95%	79.5	15.11%	53.32	49.34%	55.19	48.16%
SE	SE:MP+24P+8P+GMSR	No Censoring Applied	2	99.96%	84	17.72%	86	6.63%	28	94.90%	30	98.38%	1	100%	82	21.91%	44.71	50.96	62.79%	57.47%	33	39.89%	57	36.09%	45	37.99%	44.86	50.39%	47.98	47.73%
SE	SE:MP+ICAFIX+24P+8P+RIPTiDe	No Censoring Applied	18	91.17%	56	61.96%	36	56.25%	26	94.95%	26	98.93%	1	100%	34	80.29%	28.14	32.02	83.36%	80.99%	85	10.19%	79	17.51%	82	13.85%	55.07	48.61%	57.01	47.42%
SE	SE:MP+24P+8P	Censoring Applied	59	63.79%	87	10.31%	87	3.97%	70	71.18%	72	82.25%	2	0%	71	41.63%	64.00	72.86	39.02%	44.59%	89	0.45%	3	94.87%	46	47.66%	55.00	43.34%	59.43	46.13%
SE	SE:MP+ICAAROMA+24P	No Censoring Applied	76	22.31%	86	14.22%	82	15.40%	76	10.18%	76	10.85%	2	0%	40	76.66%	62.57	71.22	21.37%	24.43%	13	64.49%	26	70.87%	19.5	67.68%	41.04	44.53%	45.36	46.05%
SE	SE:MP+ICAAROMA+24P	Censoring Applied	75	22.36%	85	14.44%	81	15.49%	75	10.37%	75	11.14%	2	0%	39	77.55%	61.71	70.24	21.62%	24.71%	15	63.35%	27	70.80%	21	67.07%	41.36	44.35%	45.62	45.89%
SE	SE:MP+24P+8P	No Censoring Applied	60	63.78%	88	10.07%	88	3.91%	69	71.55%	69	82.61%	2	0%	72	40.93%	64.00	72.86	38.98%	44.55%	90	0.00%	4	94.05%	47	47.03%	55.50	43.00%	59.93	45.79%
SE	SE:MP+ICAAROMA+24P+8P	No Censoring Applied	56	67.01%	82	23.36%	80	20.21%	71	70.89%	71	82.57%	2	0%	62	57.25%	60.57	68.94	45.90%	52.46%	53	30.07%	47	44.50%	50	37.29%	55.29	41.59%	59.47	44.87%
SE	SE:MP+ICAAROMA+24P+8P	Censoring Applied	55	67.05%	81	23.57%	79	20.30%	72	70.87%	69	82.61%	2	0%	61	58.15%	59.86	68.12	46.08%	52.66%	55	29.05%	50	43.95%	52.5	36.50%	56.18	41.29%	60.31	44.58%
ME	ME:MP+OC+24P	Censoring Applied	89	0.06%	65	46.39%	83	14.36%	81	7.29%	81	7.36%	2	0%	56	60.20%	65.29	74.33	19.38%	22.15%	41	36.17%	18	76.36%	29.5	56.26%	47.39	37.82%	51.91	39.21%
ME	ME:MP+OC+24P	No Censoring Applied	90	0.00%	66	46.16%	84	14.28%	82	7.14%	82	7.27%	2	0%	59	59.57%	66.43	75.63	19.20%	21.95%	39	36.85%	20	75.88%	29.5	56.37%	47.96	37.79%	52.57	39.16%
SE	SE:MP+24P+8P+RIPTiDe	No Censoring Applied	7	97.73%	74	38.13%	67	30.14%	44	86.36%	49	93.26%	1	100%	66	52.06%	44.00	50.14	71.10%	66.97%	79	15.49%	90	0.00%	84.5	7.75%	64.25	39.42%	67.32	37.36%
SE	SE:MP+24P+8P+RIPTiDe	Censoring Applied	8	97.71%	73	38.32%	65	30.23%	43	86.41%	47	93.33%	1	100%	65	52.15%	43.14	49.16	71.16%	67.04%	80	14.40%	89	0.44%	84.5	7.42%	63.82	39.29%	66.83	37.23%
SE	SE:MP+24P	Censoring Applied	77	16.82%	89	0.26%	89	0.05%	77	9.55%	77	10.20%	2	0%	46	73.45%	65.29	74.33	15.76%	18.01%	57	28.77%	30	70.26%	43.5	49.51%	54.39	32.64%	58.91	33.76%
SE	SE:MP+24P	No Censoring Applied	78	16.75%	90	0.00%	90	0.00%	78	9.39%	78	9.94%	2	0%	49	72.71%	66.43	75.63	15.54%	17.76%	56	29.03%	31	70.20%	43.5	49.62%	54.96	32.58%	59.57	33.69%
